# Orexinergic projections to substantia innominata mediate arousal and analgesia

**DOI:** 10.1016/j.bja.2025.05.021

**Published:** 2025-07-08

**Authors:** Xuaner Xiang, Fei Wang, Chao Chen, Zhonghui Guan, Wei Zhou

**Affiliations:** 1 Department of Anaesthesia and Perioperative Care, University of California San Francisco, San Francisco, CA, USA; 2 Department of Ophthalmology, University of California San Francisco, San Francisco, CA, USA; 3 Department of Physiology, University of California San Francisco, San Francisco, CA, USA

**Keywords:** arousal, hypocretin, lateral hypothalamic area, neural circuit, orexin, pain, substantia innominata

## Abstract

**Background:**

Understanding neural circuits involved in anaesthesia is crucial for improving its safety and efficacy. Lateral hypothalamic area orexinergic neurones (LHA^OX^), projecting broadly, are essential in regulating arousal and pain. However, the precise targets remain unclear. Here we investigated the orexin projections to the substantia innominata (SI).

**Methods:**

Combining optogenetics, fibre photometry, electroencephalography, and electromyography allowed us to manipulate orexin activities while simultaneously recording local ligand release and global cortical activities during isoflurane anaesthesia. Brain slice electrophysiology revealed the synaptic connections in SI, while RNAscope was used to examine the distribution of orexin receptors and downstream neuronal types.

**Results:**

Presynaptic vesicles were identified in the orexin-releasing axon terminals in SI, where 49.2% of cells expressed orexin receptor-2 (OX2R) and 6.8% expressed orexin receptor-1 (OX1R). Orexin release in SI was reversibly suppressed by isoflurane. Optogenetic activation of the LHA^OX^→SI circuit increased orexin release and promoted arousal from various anaesthesia stages, including during isoflurane 0.75 vol% (*P*<0.0001), prolongation of isoflurane 3 vol% induction (*P*=0.0033), and acceleration of emergence from isoflurane 2 vol% (*P*<0.0001). Furthermore, activating this circuit induced analgesia to both thermal (*P*<0.0001) and inflammatory (*P*<0.0001) pain. Patch-clamp recordings revealed that optogenetic activation of orexin terminals in SI elicited excitatory postsynaptic currents, which were blocked by an OX2R antagonist. The SI contains more GABAergic (28.2%) and glutamatergic (12.0%) neurones than cholinergic neurones (4.1%), all of which expressed OX2R.

**Conclusions:**

*Hypothalamic area orexinergic neurones* innervate substantia innominata neurones to regulate both arousal and pain predominantly through orexin receptor-2.

**Editor’s key points**

• The neural circuits underlying anaesthesia can be resolved at high resolution using advanced neuroscience methods. Orexin has been implicated in the control of arousal from sleep and anaesthesia and in the modulation of pain, but its precise targets remain unclear.
• The authors investigated orexin projections to the substantia innominata using optogenetics, *in vivo* fibre photometry, electroencephalography, *in situ* RNA hybridisation, and electrophysiology.
• Activation of lateral hypothalamic projections to the substantia innominata aroused mice from both nonrapid eye movement sleep and isoflurane sedation, delayed induction of isoflurane anaesthesia, accelerated recovery from anaesthesia, and enhanced tolerance to pain.
• Orexinergic innervation of substantia innominata neurones regulates both arousal from anaesthesia and sleep and mediates analgesia, implicating this circuit in anaesthetic mechanisms.

Anaesthesia has been practised for close to 200 yr, yet the underlying neural circuitry remains incompletely understood. Lack of deep understanding leads to adverse events owing to unintentional anaesthetic overdosing, and neurotoxicity in both developing and ageing brains,^1^ sympathetic nervous system suppression,^2^ and prolonged postoperative recovery.^3^ Neural circuits underpin the core elements of anaesthesia: hypnosis, amnesia, immobility, and analgesia.^4^ Among these circuits, the hypothalamic orexin/hypocretin neurones, located in the lateral hypothalamic area (LHA), broadly project throughout the brain and spinal cord, playing essential roles in regulating arousal, feeding, pain perception, stress, reward, and memory formation.^5^, ^6^, ^7^, ^8^, ^9^, ^10^

Orexin neurones release the two homologous neuropeptides orexin A and orexin B, which target two G protein-coupled orexin receptor-1 (OX1R) and orexin receptor-2 (OX2R).^5^^,^^6^ Dysfunction of the orexin circuit is linked to narcolepsy with cataplexy, characterised by disrupted sleep–wake patterns and sudden loss of muscle tone.^11^^,^^12^ Studies have shown that OX2R knockout mice exhibit symptoms of narcolepsy, whereas OX1R knockout mice are grossly normal, highlighting the critical role of OX2R in sleep–wake control.^13^ However, the locus coeruleus (LC), rich in OX1R, is well-known for its adrenergic neurones and their wake-promoting role. Orexin projections serve diverse functions; for example, the projection to the lateral habenula is involved in regulating aggressive behaviour in male mice,^14^ whereas the projection to the ventral tegmental area (VTA) has a role in regulating stress-induced cocaine recurrence.^15^ Animal experiments showed that orexin is involved in various aspects of anaesthesia, including emergence, airway patency, autonomic tone, and gastroenteric motility.^16^, ^17^, ^18^, ^19^ The precise orexin circuits responsible for arousal from anaesthesia are not fully understood.

Beyond arousal control, orexin also plays a crucial role in modulating analgesia, an essential component of anaesthesia.^20^^,^^21^ Given its widespread projections throughout the brain and spinal cord, orexin contributes to pain modulation at multiple levels. At the spinal cord level, orexin innervates all segments from cervical to sacral, and directly activates the dorsal root ganglia cells through OX1R.^22^^,^^23^ In the brain, orexin regulates pain via key regions such as the rostral ventromedial medulla,^24^ VTA,^25^ and periaqueductal grey,^26^ involving various neurotransmitters including dopamine, adenosine, endocannabinoids, and opioid peptides. Nevertheless, the specific circuitry involving orexin neurones that relieves pain is yet to be determined.

Previously, we mapped the 3D projections of orexin neurones in the whole mouse brain, revealing dense projections to the substantia innominata (SI),^27^ located at the anteromedial and ventral part of the cerebral hemisphere. The SI is involved in aggressive response, cortical processing, and sleep.^28^, ^29^, ^30^ It contains the nucleus basalis of Meynert, a major source of cholinergic input to the cerebral cortex. Cholinergic output has been known to be a key for cortical activation and anaesthesia arousal.^31^^,^^32^ The SI also contains significant populations of GABAergic and glutamatergic neurones.^33^ Beyond arousal control, it is part of an affective pain circuit and receives innervation from the central amygdala (CeL) to modulate fear-related behaviours.^34^ Given that the LHA responds to noxious stimuli and regulates pain processing,^35^^,^^36^ important questions persist regarding the synaptic connections between LHA^OX^ and SI, the distribution of the two orexin receptors, and the profile of neuronal types innervated by orexinergic terminals. In this study, we used optogenetics, EEG, EMG, *in vivo* fibre photometry, brain slice electrophysiology, and RNAscope *in situ* hybridisation to investigate the LHA^OX^→SI circuit, and its role in arousal and pain regulation.

## Methods

All experimental procedures were approved by the Institutional Animal Care and Use Committee, University of California, San Francisco. Mice were maintained in a strictly controlled environment with *ad libitum* access to food and water. The light cycle starts at 07:00 and ends at 19:00. Temperature was controlled at 20–22°C.

We used 157 mice aged 6–16 weeks and weighing 18–32 g. The Orexin-Cre-2A-EGFP line was a gift from Akihiro Yamanaka (Nagoya University, Nagoya, Japan),^8^ and was bred onto the C57BL/6 genetic background. Wild-type C57/BL6 mice were purchased from Jackson Laboratories (stock No: 000664). We used the following numbers of Orexin-Cre mice: 45 in the experiments on nonrapid eye movement (NREM) sleep, arousal, and emergence behaviour with optogenetic stimulation and EEG and EMG recording; 26 in the experiments on orexin release during anaesthesia sedation, induction, and emergence with optogenetic stimulation and fibre photometry; 42 for the hot plate and formalin tests; 16 for investigating SI and LHA^OX^ neuronal activity changes during different anaesthesia doses with optogenetic stimulation, fibre photometry, and EEG and EMG recording; 13 for brain slice recordings; and 10 in the neural tracing experiments. We used five wild-type mice for the RNAscope experiments. Only male mice were used to avoid hormonal fluctuations associated with the oestrous cycle. The orexin promoter is X-linked, so male Orexin-Cre mice all contain the same number of Cre and orexin genes.

### Virus injection

AAV1-phSyn1(S)-FLEX-tdTomato-T2A-SypEGFP-WPRE (51509-AAV1), AAVrg-hSyn-DIO-mCherry (50459-AAVrg), AAV-EF1a-double floxed-hChR2(H134R)-mCherry-WPRE-HGHpA (20297-AAVretro), AAV-Syn-DIO-ChrimsonR-tdTomato (62723-AAV5), AAV5-CAG-FLEX-rc [Jaws-KGC-GFP-ER2] (84445-AAV5), AAV1-Syn-GCaMP6s-WPRE-SV40 (100843-AAV1), and AAV1-Syn-Flex-GCaMP6f-WPRE-SV40 (100833-AAV1) were purchased from Addgene (Watertown, MA, USA). The AAV2-Oxlight1 plasmid (169792; Addgene) was purchased from Addgene, and the virus was homemade according to the published protocol from the Salk Institute (https://www.salk.edu/science/core-facilities/viral-vector-core/services/). All AAV viruses were aliquoted and stored at –80°C. Each virus aliquot was not re-frozen after thawing.

Intracranial injections were performed under balanced anaesthesia using a robotic stereotaxic instrument (Neurostar, Tubingen, Germany). Meloxicam (5 mg kg^–1^ s.c.), ampicillin (10–20 mg kg^–1^ s.c.), buprenorphine (0.1 mg kg^–1^ s.c.), bupivacaine 0.25% (0.025 ml s.c.), and isoflurane (Henry Schein Animal Health, Dublin, OH, USA) were given during the procedure. A 2 μl Hamilton™ Neuros™ 7000 Series Modified Microliter Syringe with Knurled Hub Needle (14-815-903; Hamilton Company, Reno, NV, USA) was used for injections, and 0.5 μl virus was delivered over 10 min. The needle was left *in situ* for 5 min and slowly withdrawn. The skin was stapled after injection surgery using the BD Autoclip™ Wound Closing System (22-275998; Fisher Scientific, South San Francisco, CA, USA). Injection coordinates were: lateral hypothalamic area (AP, –1.46 mm, ML, within 0.95 mm, and DV, 5.1–5.0 mm), SI (AP, –0.6 mm, ML, within 1.75 mm, and DV, 4.8–4.7 mm).

### Fibre photometry

For optogenetic and EEG experiments, mice were implanted with bilateral optical fibres (400 μm core, 0.39 N.A.; R-FOC-BL400C-39NA; RWD Life Science, Shenzhen, Guangdong, China) into the SI (AP, –0.6 mm, ML, within 1.75 mm, and DV, 4.7 mm). For fibre photometry, all mice were implanted with unilateral optical fibre (200 μm core, 0.39 N.A.; R-FOC-BL200C-39NA; RWD Life Science) into the left side of the SI (AP, –0.6 mm, ML, within 1.75 mm, and DV, 4.7 mm).

### Electroencephalography and electromyography electrodes

After receiving bilateral stereotaxic viral injections and optical fibre implantation, mice received EEG and EMG headmount (8201; Pinnacle Technology, Lawrence, KS, USA) implantation during the same procedure. A 23-G surgical needle was used to drill four guide holes, including two frontal cortical areas (AP, 1 mm, ML, within 1.25 mm) and two parietal areas (AP, –3 mm, ML, within 2.5 mm). Four screws with wires (8493, Pinnacle Technology) were placed into the skull through the holes. The wires were then soldered onto a six-pin connector headmount. EMG leads were placed into the neck muscle. The headmount was secured with dental cement (C&B Metabond® Quick Adhesive Cement System; Parkell, Edgewood, NY, USA) onto the skull. Behavioural experiments were conducted 4 weeks after surgery to allow sufficient recovery.

### Electroencephalography recording with optogenetic manipulation

Mice were anaesthetised with isoflurane 3 vol% with O_2_ flow of 4 L min^–1^ for 1 min in an induction chamber and then attached to the EEG/EMG system (8200-K1-SL 2 EEG/1 EMG Mouse System for Sleep; Pinnacle Technology), with the bilateral optical fibres through the black ceramic ferrule mating sleeves (R-MS-1.25; RWD Life Science Inc). The bifurcated fibre bundle (Ø400 μm core, 0.39 NA, FC/PC to Ø1.25 mm Ferrules, 1 m Long; BFYL4LF01; Thorlabs, Newton, NJ, USA) was connected to a 473 nm laser stimulator (Aurora-200; Newdoon, Hangzhou, Zhejiang, China) or a 635 nm red laser stimulator (Intelligent Optogenetics System IOS-635; RWD Life Science Inc.). The mice were acclimated for 24 h before EEG-related behavioural testing, and during each testing session they were given another 30 min of acclimation before data collection. Optogenetic stimulation parameters were based on our previous study.^27^

### Fibre photometry with optogenetic manipulation

Mice were anaesthetised with isoflurane 3 vol% with O_2_ flow of 4 L min^–1^ for 1 min in an induction chamber, and a low-autofluorescence mono-fibreoptic patch cord (MFP_400/440/3000-0.37_1m_FCM-MF1.25(F)_LAF, Doric Lenses Inc. Québec, Canada) was connected to the R821 tricolour multichannel fibre photometry system (RWD Life Science Inc.). The green fluorescence of calcium signals was evoked by 470 nm, and 410 nm was used to acquire reference signals to eliminate noise. The 635 nm red laser stimulator was connected to the R821 fibre photometry system to manipulate neuronal activity in mice expressing ChrimsonR opsin and record the calcium activities simultaneously. Mice were given >30 min of acclimation before data collection. The parameters of optogenetic activation are described below in the behavioural tests.

### Immunohistochemistry

Mice were transcardially perfused 4–8 weeks after virus injection with cold phosphate-buffered saline (PBS, 137 mM NaCl, 2.7 mM KCl, 10 mM Na_2_HPO_4_, 1.8 mM KH_2_PO_4_, pH 7.4) followed by a cold solution of 4% paraformaldehyde (PFA, Electron Microscopy Sciences, Hatfield, PA, USA) in PBS. Brains were removed and post-fixed in 4% PFA for 6 h at 4°C before being transferred to 30% sucrose for at least 2 days of dehydration until the tissue stayed afloat. A series of 35 μm coronal–sagittal brain slices were sliced using a cryostat (Leica CM3050S, Deerfield, IL, USA).

For immunohistochemistry staining, brain slices were blocked with blocking solution (5% donkey serum, 3% bovine serum albumin, and 0.3% Triton-X100 in PBS) for 1 h at room temperature, followed by incubation with primary antibodies at 4°C for 16–24 h. After brain slices were washed three times with PBS (10 min each time), the slices were incubated with secondary antibodies for 2 h at room temperature, followed by washing three times. Finally, the slices were covered with DAPI Fluoromount-G mounting medium (0100-20, SouthernBiotech, Birmingham, AL, USA). Antibodies were diluted in a blocking solution as follows. mCherry: primary, chicken anti-mCherry (1:400, NBP2-25158; Novus Biologicals, Centennial, CO, USA); secondary, Cy3-conjugated AffiniPure goat anti-chicken IgY++ (1:400, 103-165-155; Jackson Labs, Sacramento, CA, USA). cFos: primary, rabbit anti-cFos (1:100, 2250S, Cell Signaling, Danvers, MA, USA); secondary, Alexa Fluor 488-conjugated donkey anti-rabbit IgG (H + L) (1:400, 711-546-152; Jackson Labs). Confocal images were taken with a Leica TCS SP8. Tile scans were facilitated with Leica Application Suite software.

### Isoflurane arousal test

Optogenetic manipulation mice were anaesthetised with isoflurane 3 vol% for 1 min in an induction chamber. After being quickly attached to the instruments, mice were placed in a clear isoflurane chamber with optical fibres exiting through a sealed port. After 30 min of acclimation, the chamber was equilibrated with isoflurane 3 vol% for 1 min, then changed to isoflurane 0.75 vol% for 3 min before a 20 Hz, 20 ms light pulse (473 or 635 nm) was applied for 30 s. The isoflurane was continued for another 1 min after stimulation and switched to 100% O_2_ to let the mice recover. The time from the laser on to the point when the mice started to emerge (moving, kicking, or tail rising) was recorded as the latency to wake. The trials were repeated three times on the same day from 09:00 to 18:00. Control group mice received the same treatments. All trials were age-matched with similar animals. All anaesthesia chambers were placed on a temperature-controlled heating pad to regulate body temperature at 35–37°C.

### Isoflurane induction test

Mice were connected to the photometry system and placed in a clear isoflurane chamber with optical fibres exiting through a sealed port. Photometry data were collected 10 min before the induction test as a baseline. Mice were treated with 20 Hz, 20 ms light pulse (635 nm), 1 s on, 1 s off, from 5 min before the induction test to the end. Mice were treated with isoflurane 3 vol% for 3 min. The time from the onset time of isoflurane to the point when the mice lost righting reflex was recorded as loss of righting reflex (LoRR). The trials were conducted from 09:00 to 18:00 and repeated two times with 1-day intervals. All trials were age-matched with similar animals. All anaesthesia chambers were placed on a temperature-controlled heating pad to regulate body temperature at 35–37°C.

### Isoflurane emergence test

Mice received isoflurane 2 vol% for 30 min in the induction chamber, and then were quickly attached to the optical fibres and treated with or without optical stimulation (20 Hz, 20 ms pulse width, 1 s on, 1 s off) in a clear acrylic chamber open to the room air. They were placed on their backs to test how quickly the righting reflex returned (return of the righting reflex [RoRR]), when the animal stood on all four paws, which was used to indicate emergence from anaesthesia. The emergence time from turning off isoflurane to RoRR was recorded for analysis. Trials were performed from 09:00 to 18:00 and repeated three times at 3-day intervals. The isoflurane induction chamber was placed on a temperature-controlled heating pad. A temperature probe connected to a temperature controller was placed underneath the animal body to regulate body temperature at 35–37°C. The control group received the same treatments. All trials were age-matched with similar animals.

### Hot plate test

Mice were anaesthetised in the induction chamber with isoflurane 3 vol% for 1 min to facilitate optical fibre attachment. Then mice went through 5 min of optical stimulation with or without the laser (20 Hz, 20 ms pulse width, 1 s on, 1 s off), followed by the hot plate test. An acrylic container was used as an enclosure to prevent the animal from escaping. The temperature used in the hot plate test was 55°C. Mice were placed on the hot plate while continuing optical stimulation until the animals showed licking, fanning, or jumping, at which point the mice were immediately removed from the hot plate, the paw withdrawal latency was recorded for analysis. Mice were removed if there was no response within 45 s. The trials were repeated three times on the same day from 09:00 to 15:00, at 60-min intervals, and again repeated two more times at 3-day intervals. The control group received the same treatments. All trials were age-matched with similar animals.

### Formalin test

With isoflurane 3 vol% for 1 min of anaesthesia, mice were injected with 10 μl of 5% formalin or saline under the skin of the dorsal surface of the left hind paw. Mice were placed onto the recording platform, and each animal was individually placed in an acrylic cylinder with a mirror underneath. Then mice were treated with 60 min of optical stimulation with the laser (20 Hz, 20 ms pulse width, 1 s on, 1 s off), and behaviour was videotaped for 60 min after the mice were placed on the recording platform. The video was analysed later and licking time of the left hind paw was recorded, with 0–15 min considered as the acute phase and 15–60 min considered the chronic phase.

### Electrophysiology

The 8- to 12-week-old Orexin-Cre mice were anaesthetised with isoflurane and transcardially perfused with ice-cold cutting solution containing 89.5 mM choline chloride, 20.6 mM Tris, 2.5 mM KCl, 1.2 mM NaH_2_PO_4_, 20 mM HEPES, 5 mM sodium ascorbate, 2 mM thiourea, 3 mM sodium pyruvate, 25 mM glucose, 30 mM NaHCO_3_, 10 mM MgSO_4_, 0.5 mM CaCl_2_, and 300–310 mOsm, adjusted to pH 7.4 with HCl. The solution was bubbled with carbogen (95% O_2_/5% CO_2_). Mice were decapitated, and the brain was collected and cut into 250 μm thick sections with a vibratome (Leica VT1000S). The brain slices were recovered in cutting solution for 10–15 min at 34°C, then incubated in HEPES artificial cerebrospinal fluid (aCSF) recovery solution, which consisted of 92 mM NaCl, 2.5 mM KCl, 1.25 mM NaH_2_PO_4_, 30 mM NaHCO_3_, 20 mM HEPES, 25 mM glucose, 2 mM thiourea, 5 mM Na-ascorbate, 3 mM Na-pyruvate, 2 mM CaCl_2_, 2 mM MgSO_4_, and 300–310 mOsm, adjusted to pH 7.3–7.4 with HCl. After incubation for 45–60 min at room temperature, the brain slice was transferred to the recording chamber and perfused (3 ml min^–1^) with recording aCSF, which consisted of 124 mM NaCl, 2.5 mM KCl, 1.2 mM NaH_2_PO_4_·2H_2_O, 24 mM NaHCO_3_, 5 mM HEPES, 12.5 mM glucose, 2 mM CaCl_2_, 2 mM MgSO_4_, and 300–310 mOsm, adjusted with NaOH to pH 7.3–7.4. All external solutions were saturated with 95% O_2_, 5% CO_2_.

The recording glass pipettes (BF150-86-10; Sutter Instrument, Novato, CA, USA) were pulled by a micropipette puller (P1000, Sutter Instrument) into a recording electrode (3–5 MΩ). The recording electrode was filled with a potassium-based internal solution containing 145 mM potassium gluconate, 10 mM HEPES, 1 mM EGTA, 2 mM Mg-ATP, 0.3 mM Na_2_-GTP, and 2 mM MgCl_2_, 290–300 mOsm adjusted to pH 7.2–7.3 with KO. Neurones were identified and subjected to electrophysiological recordings with a 60x water-immersion lens (Zeiss, Dublin, CA, USA). The recordings were performed using whole-cell techniques (MultiClamp 700B Amplifier, Digidata 1322A analog-to-digital converter; Molecular Devices, San Jose, CA, USA) and pClamp 9.2 software (Axon Instruments/Molecular Devices). The traces were digitised at 10kHz, and the data were analysed using pClamp/Clampfit.

Optogenetic stimulation was performed using a 635 nm laser (Intelligent Optogenetics System IOS-635; RWD Life Science Inc) using 20 Hz and 20 ms light pulses per trial, five trials per neurone. After breaking into the neurones, neurones were held at –65 mV to record excitatory postsynaptic currents (EPSCs), and 0 mV to record inhibitory postsynaptic currents (IPSCs). Voltage-clamp experiments were conducted to record EPSCs and IPSCs, and current clamp tests were performed to record action potentials. Furthermore, 10 μM SB-334867 (SML1530; Millipore Sigma), and 30 μM TCS OX2 29 HCl (SML2879, Millipore Sigma) were added to aCSF perfusion solutions to block OX1R and OX2R separately.

### RNAscope *in situ* hybridisation

C57/BL6 mice (14–16 weeks) were transcardially perfused as described above, brains were post-fixed at 4°C in 30% sucrose for 24 h, and cut into 15 μm. Slides were stored at –80°C until staining. Staining was performed with the RNAscope Multiplex Fluorescent Detection Reagents v2 kit (323110; Advanced Cell Diagnostics, Newark, CA, USA) according to the user manual. Probes for OX1R (Hcrtr1, Cat No. 466631-C1), ChAT (CHAT, Cat No. 408731-C1), vGlut2 (Slc17a6, Cat No. 428871-C2), vGAT (Slc32a1, Cat No. 319191-C2), TH (TH, Cat No. 317621-C2), and OX2R (Hcrtr2, Cat No. 581631-C3) were commercially available from Advanced Cell Diagnostics. In brief, slides were post-fixed in 4% PFA for 2 h and briefly washed in PBS. After being treated with H_2_O_2_ for 10 min at room temperature, slides were washed in diethylpyrocarbonate (DEPC)-treated double-distilled water (ddH_2_O), and boiled in Target Retrieval solution (98–102°C) for 5 min. After a brief washing in water and dehydration in absolute ethanol, slides were dried at 60°C incubator for 5 min, they were moistened with ddH_2_O, and incubated with protease Ill for 15 min at 40°C in the HybEZ oven (Advanced Cell Diagnostics). Slides were washed again in ddH_2_O and hybridised with the mixture of probes in different channels for 2 h in a HybEZ oven at 40°C. Subsequently, the hybridisation was amplified with AMP-1 for 30 min, AMP-2 for 30 min, and AMP-3 for 15 min, respectively. The signals were then developed for each channel by incubating with HRP-C1 for 15 min, the Opal™ 520 Reagent Pack (FP1487001KT, Akoya Biosciences Inc, Marlborough, MA, USA) for 30 min, and HRP blocker for 15 min; HRP-C2 for 15 min, the Opal™ 620 Reagent Pack (FP1495001KT, Akoya Biosciences Inc) for 30 min, and HRP blocker for 15 min; HRP-C3 for 15 min, Opal™ 690 Reagent Pack (FP1497001KT, Akoya Biosciences Inc) for 30 min, and HRP blocker for 15 min. All amplification and development were performed at 40°C in the HybEZ oven, and slides were washed using an ACD wash buffer after each step. Next, the slides were mounted with DAPI Fluoromount-G mounting medium (0100-20, SouthernBiotech), and covered with coverslips. Imaging was performed with a confocal laser scanning microscope Leica TCS SP8 with a 40x oil immersion objective. The RNAscope images were manually adjusted with ImageJ/Fiji and the data were automatically analysed with the QuPath V0.5.1 (https://qupath.readthedocs.io/).

### Data analysis and statistics

Electrophysiology data were analysed with Clampfit 10 (Molecular Devices). EPSC and IPSC amplitudes were calculated as the difference between the peak amplitude in a predefined window after light stimulation onset and mean amplitude just preceding the EPSC or IPSC. EPSC or IPSC latency was measured as the time from the onset of laser stimulation to the first intersection between the baseline of EPSC or IPSC, which can be easily identified at the place of maximal increasing/decreasing curvature. EEG data were analysed with MATLAB (R2023B; MathWorks, Natick, MA, USA). Four EEG frequency bands for power analysis were delta, 0.5–4 Hz; theta, 4–8 Hz; alpha, 8–12.0 Hz; and beta, 12–30 Hz. Power spectral density (PSD) with 95% confidence intervals (CIs) and burst suppression ratio (BSR) were calculated and plotted with MATLAB. Fibre photometry data were processed with the RWD fibre photometry software. The figures were created using Prism 10.3.1(GraphPad Software Inc. San Diego, CA, USA), MATLAB, ImageJ, QuPath, and Illustrator (Adobe, San Jose, CA, USA).

Statistical details are presented in the figure legends. All data were analysed using Prism 10.3.1. Two-tailed, unpaired *t*-test was used to analyse the significance between two groups with normally distributed data. Ordinary one-way analysis of variance (anova) was used to compare the significance of three or more groups with one independent variable. Two-way anova was used to compare the mean differences between groups with two independent variables. anova was followed by *post hoc* tests with multiple comparison corrections, as indicated in the figure legends. All data are shown as mean (sem).

## Results

### Hypothalamic orexin neurones project to the substantia innominata

**Fig 1:**
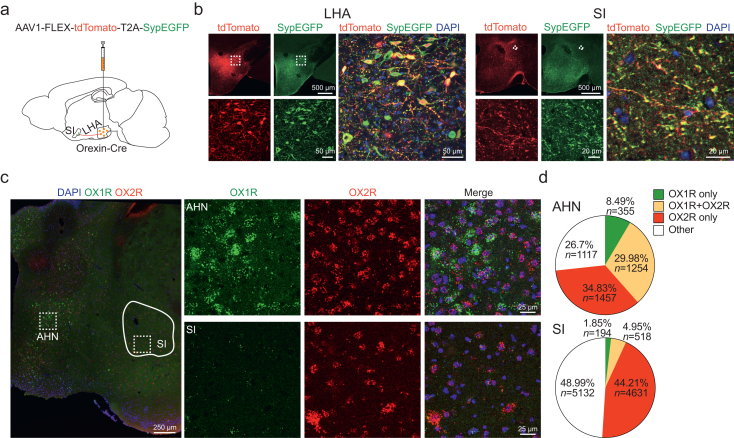
Hypothalamic orexin neurones project to the substantia innominata (SI). (a) AAV1-FLEX-tdTomato-T2A-SypEGFP was injected into the lateral hypothalamic area (LHA) of Orexin-Cre mice. (b) Orexin cell bodies in the LHA and axon terminals in the SI are seen by tdTomato and synaptophysin-EGFP (SypEGFP). (c) Images of RNAscope *in situ* hybridisation in the anterior hypothalamic nucleus (AHN) and SI. (d) Distributions of orexin receptor-1 (OX1R) and orexin receptor-2 (OX2R) in AHN and SI. AHN, six slices from four brains. SI, ten slices from five brains.

Our previous results revealed dense fibres observed in the SI.^27^ To confirm the presence of orexin terminals in SI, we utilised the synaptic vesicle protein marker synaptophysin (Syp). We injected adeno-associated virus (AAV)-FLEX-tdTomato-T2A-SypEGFP into the LHA of Orexin-Cre mice (Fig. 1a) containing the T2A sequence that encodes a self-cleaving peptide that separates SypEGFP from tdTomato. This targets SypEGFP to presynaptic vesicles, while tdTomato is expressed throughout the cell. Punctate SypEGFP expression was observed in both LHA and SI (Fig. 1b), suggesting the presence of orexinergic synaptic terminals in these regions.

We analysed the distribution of postsynaptic orexin receptors in SI to confirm the innervation by orexin terminals. Using RNAscope *in situ* hybridisation to probe brain slices for OX1R and OX2R, mRNAs revealed 44.2% of SI cells expressing OX2R only, 1.9% OX1R only, and 5.0% expressing both receptors (Fig. 1c and d). The distributions of OX1R and OX2R are highly variable in the brain. The paraventricular nucleus of the thalamus (PVT) expresses both OX1R and OX2R, whereas the LC primarily expresses OX1R.^9^^,^^10^ Our RNAscope data confirmed this by showing similar staining of OX1R and OX2R mRNA in the PVT and predominantly OX1R mRNA in the LC (Supplementary Fig. 1). In the nearby anterior hypothalamus nucleus (AHN) region of the same brain slice, the distribution of two receptors was dramatically different, with a higher proportion of OX1R-only cells (8.5%) compared with 1.9% in SI (Fig. 1c and d). Of the AHN cells, 30.0% were double-labelled for OX1R and OX2R, in contrast to 5.0% in the SI. These results validated our RNAscope data, showing that about half of SI cells express orexin receptors, with OX2R being the predominant type. This suggests potential innervation of SI neurones by orexinergic terminals.

### Local release of orexin in the substantia innominata during anaesthesia

**Fig 2:**
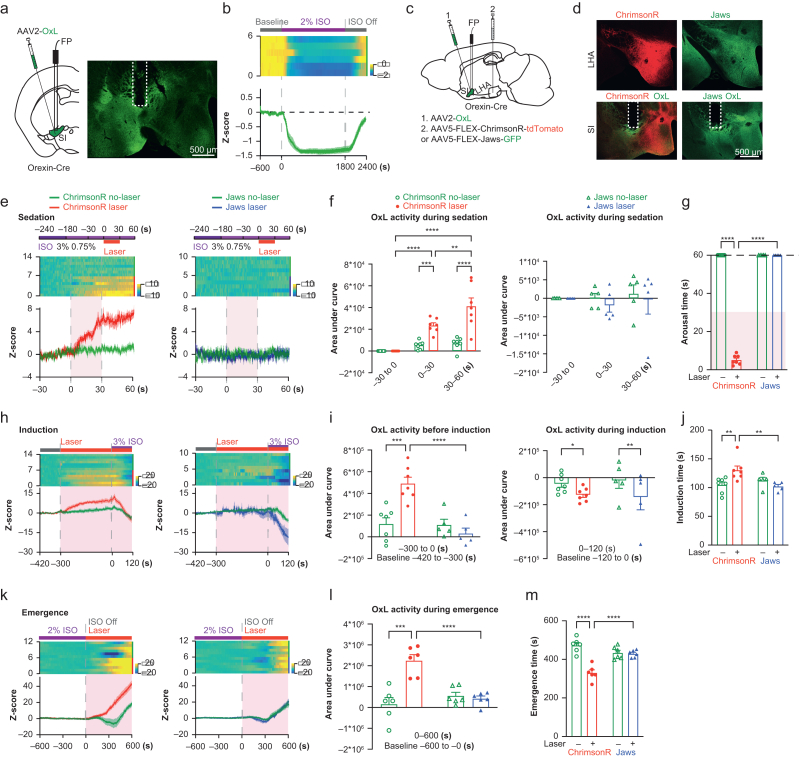
Activating the LHA^OX^→SI projection increases orexin release and influences different anaesthesia levels. (a) AAV2-OxLight1 was injected into the SI, and an optical fibre was implanted above. (b) OxLight1 (OxL) signal changes during isoflurane anaesthesia. (c and d) In addition to AAV2-OxLight1 injection and fibre implantation into the SI, AAV5-FLEX-ChrimsonR-tdTomato or AAV5-FLEX-Jaws-GFP was injected into the ipsilateral LHA. (e) OxL signals during isoflurane 0.75 vol% sedation. (f) AUC analysis of OxL signals during isoflurane sedation. ChrimsonR groups: two-way anova, effect of laser stimulation: *F*_(1, 12)_=29.34, *P*=0.0002, effect of time: *F*_(2, 24)_=29.30, *P*<0.0001, interaction between laser stimulation and time: *F*_(2, 24)_=14.65, *P*<0.0001, Tukey’s multiple comparison test, *n*=7. Jaws groups: two-way anova, effect of laser stimulation: *F*_(1, 8)_=0.3878, *P*=0.55, effect of time: *F*_(2, 16)_=0.25, *P*=0.79, interaction between laser stimulation and time: *F*_(2, 16)_=0.14, *P*=0.87; Tukey’s multiple comparison test, *n*=5. (g) Arousal times from laser stimulation during isoflurane sedation. The dashed line indicates the cut-off point at 60 s after laser activation. Two-way anova, effect of groups: *F*_(1, 10)_=2259, *P*<0.0001, effect of laser stimulation: *F*_(1, 10)_=2259, *P*<0.0001, interaction between groups and laser stimulation: *F*_(1, 10)_=2259, *P*<0.0001; uncorrected Fisher’s LSD analysis, ChrimsonR group, *n*=7; Jaws group, *n*=5. (h) OxL signals during isoflurane 3 vol% induction. (i) AUC analysis of OxL signals. Pre-induction: two-way anova, effect of groups: *F*_(1, 10)_=16.52, *P*=0.0023, effect of laser stimulation: *F*_(1, 10)_=6.58, *P*=0.028, interaction between groups and laser stimulation: *F*_(1, 10)_=15.69, *P*=0.003; uncorrected Fisher’s LSD analysis. Post-induction: two-way anova, effect of groups: *F*_(1, 10)_=0.004, *P*=0.954, effect of laser stimulation: *F*_(1, 10)_=18.83, *P*=0.002, interaction between groups and laser stimulation: *F*_(1, 10)_=0.771, *P*=0.401; uncorrected Fisher’s LSD analysis, ChrimsonR group, *n*=7; Jaws group, *n*=5. (j) Induction time comparison. Two-way anova, effect of groups: *F*_(1, 10)_=2.405, *P*=0.152, effect of laser stimulation: *F*_(1, 10)_=2.164, *P*=0.172, interaction between groups and laser stimulation: *F*_(1, 10)_=12.04, *P*=0.006; uncorrected Fisher’s LSD analysis, ChrimsonR group, *n*=7; Jaws group, *n*=5. (k) OxL signals during isoflurane 2 vol% emergence. (l) AUC analysis of OxL signals during emergence. Two-way anova, effect of groups: *F*_(1, 10)_=9.168, *P*=0.013, effect of laser stimulation: *F*_(1, 10)_=16.87, *P*=0.002, interaction between groups and laser stimulation: *F*_(1, 10)_=21.82, *P*=0.001; uncorrected Fisher’s LSD analysis, *n*=6 for both groups. (m) Emergence times. Two-way anova, effect of groups: *F*_(1, 10)_=4.503, *P*=0.06, effect of laser stimulation: *F*_(1, 10)_=33.95, *P*=0.0002, interaction between groups and laser stimulation: *F*_(1, 10)_=29.64, *P*=0.0003; uncorrected Fisher’s LSD analysis, *n*=6 for both groups. ∗*P*≤0.05, ∗∗*P*≤0.01, ∗∗∗*P*≤0.001, ∗∗∗∗*P*≤0.0001. All data expressed as mean (sem). anova, analysis of variance; AUC, area under the curve; FP, fibre photometry; LHA^OX^, lateral hypothalamic area orexinergic neurones; LSD, least significant difference; sem, standard error of the mean; SI, substantia innominata.

To determine whether the local release of orexin participates in anaesthesia, we used *in vivo* fibre photometry using the recently developed orexin sensor OxLight1, which was engineered by inserting a circularly permutated green fluorescent protein (GFP) into the human OX2R.^37^ The non-Cre-dependent AAV2-OxLight1 virus was injected into the SI and optical fibre was implanted just above the injection site (Fig. 2a). To visualise how orexin release responds to anaesthetics, we exposed mice to isoflurane 2 vol%, a standard anaesthetic dose used for rodent surgery. Upon exposure, the OxLight1 signal in the SI quickly decreased alongside behavioural arrest and stayed low until termination of isoflurane, after which a marked increase in the OxLight1 signal was observed accompanied by arousal behaviour (Fig. 2b). These findings demonstrated that suppression and recovery of orexin release in the SI are closely associated with anaesthesia-induced behavioural changes.

### Activating the LHA^OX^→SI projection increases orexin release and reverses anaesthesia sedation

To explore the relationship between orexin release and anaesthesia behaviour, we combined optogenetics and fibre photometry. Specifically, we used ChrimsonR, a red light-drivable channelrhodopsin (cation channel),^38^ and Jaws, a red light-drivable cruxhalorhodopsin (chloride pump),^39^ to modulate orexinergic activity in the SI. Meanwhile, we recorded orexin release dynamics at the same location. This is achieved by injecting excitatory AAV5-FLEX-ChrimsonR-tdTomato or inhibitory AAV5-FLEX-Jaws-GFP into the LHA, together with AAV2-OxLight1 into the SI (Fig. 2c and d). An optical fibre was implanted just above the SI, allowing stimulation of orexin terminals through the 635 nm channel while simultaneously recording the OxLight1 signals through the 473 nm channel.

Different types of anaesthesia, from light sedation to deep general anaesthesia, involve a spectrum of brain states, tailored for specific procedures to provide the best patient comfort but with minimised anaesthetic exposure. We first examined orexin release during light sedation. Anaesthesia was induced with isoflurane 3 vol% for 1 min, then maintained at 0.75 vol% for 3 min followed by activation of the ChrimsonR or Jaws for 30 s (635 nm, 20 Hz, 20 ms; Fig. 2e). Laser stimulation in the ChrimsonR group (F_(1, 12)_=29.34, *P*=0.0002) to activate ChrimsonR increased the OxLight1 signal compared with the no-laser control (0–30 s: 23400 [2500] *vs* 5340 [1420], Tukey’s multiple comparisons, *P*=0.0007; *n*=7), and the OxLight1 signal remained elevated after stimulation ended (30–60 s: 41 200 [7620] *vs* 6950 [2190]; Tukey’s multiple comparisons, *P*<0.0001; *n*=7; Fig. 2e and f), also using two-way anova. All animals displayed robust arousal behaviour (latency to wake: 5.1 [1.0] s, two-way anova, *F*_(1, 10)_=2259, *P*<0.0001, uncorrected Fisher’s least significant difference [LSD], *P*<0.0001; *n*=7) following ChrimsonR activation (Fig. 2g). In contrast, no significant changes in OxLight1 signals were observed in the ChrimsonR no-laser control (–30 to 0 s *vs* 0–30 s: *P*=0.469, –30 to 0 s *vs* 30–60 s: *P*=0.285, and 0–30 s *vs* 30–60 s: *P*=0.931), or the Jaws groups with or without laser stimulation (two-way anova, *F*_(1, 8)_=0.3878, *P*=0.55, *n*=5; Fig. 2e and f). Uncorrected Fisher’s LSD showed no difference in arousal behaviour in the Jaws group with or without laser stimulation (*P*>0.9999), and comparisons between ChrimsonR and Jaws group showed no difference in the no-laser condition (*P*>0.9999), but a significant difference in the laser condition (*P*<0.0001, uncorrected Fisher’s LSD, *n*=5; Fig. 2g).

To verify the inhibitory function of Jaws, we tested Jaws in the acute brain slice. Whole-cell patch recordings showed that Jaws activation hyperpolarised membrane potential and blocked evoked action potentials (Supplementary Fig. 2a and b). These findings showed that activation of orexin terminals in the SI elicited release of orexin peptides and reversed the sedation by isoflurane.

### LHA^OX^→SI activity influences the rates of anaesthesia induction and emergence

We examined how orexin activity affects induction and emergence of general anaesthesia. Recognising the importance of time needed for orexin release to reach a plateau, we introduced a 5-min opto-stimulation (–300 to 0 s) before initiating isoflurane 3 vol% (Fig. 2h). Two-way anova analysis (*F*_(1, 10)_=15.69, *P*=0.0027, uncorrected Fisher’s LSD) showed that stimulating orexin terminals via ChrimsonR in the SI caused a sustained elevation of the OxLight1 signal before isoflurane exposure (ChrimsonR laser *vs* ChrimsonR no-laser: 491 000 [58 700] *vs* 119 000 [56700], *P*=0.0005; *n*=7). In contrast, Jaws activation did not further reduce the OxLight1 signal, likely owing to the low basal activity of orexin neurones (Jaws laser *vs* Jaws no-laser: 31 200 [49 500] *vs* 111 000 [51 700], *P*=0.3825; *n*=5). Comparisons between ChrimsonR and Jaws show no difference under the no-laser condition (uncorrected Fisher’s LSD, *P*=0.9192), but a significant difference under the laser condition (uncorrected Fisher’s LSD, *P*<0.0001; Fig. 2i).

Exposure to isoflurane 3 vol% (0–120 s) decreased the OxLight1 signal in both the ChrimsonR group and Jaws group (two-way anova, *F*_(1, 10)_=0.771, *P*=0.401, ChrimsonR laser *vs* ChrimsonR no-laser: –127 000 [19 000] *vs* –45 500 [25 600], uncorrected Fisher’s LSD, *P*=0.023, *n*=7; Jaws laser *vs* Jaws no-laser: –144 000 [94 500] *vs* –20 900 [58 000], uncorrected Fisher’s LSD, *P*=0.0066, *n*=5) (Fig. 2h and i). Comparisons between ChrimsonR and Jaws showed no difference in OxLight1 signal drops in the no-laser condition (uncorrected Fisher’s LSD, *P*=0.7299) and the laser condition (uncorrected Fisher’s LSD, *P*=0.8143; Fig. 2i). Two-way anova analysis of induction times (*F*_(1, 10)_=12.04, *P*=0.006) showed the ChrimsonR activation significantly prolonged induction time, as indicated by the time to LoRR (ChrimsonR laser *vs* ChrimsonR no-laser: 130 [7] *vs* 105 [5] s, uncorrected Fisher’s LSD, *P*=0.0033, *n*=7; Fig. 2j). However, no significant change in induction time was observed in the Jaws group (Jaws laser *vs* Jaws no-laser: 102 [3] *vs* 112 [6] s, uncorrected Fisher’s LSD, *P*=0.2201, *n*=5; Fig. 2j). Comparisons between ChrimsonR and Jaws groups showed no significant difference in the no-laser condition (uncorrected Fisher’s LSD, *P*=0.3626), but a significant difference in the laser condition (uncorrected Fisher’s LSD, *P*=0.0031; Fig. 2j). The results suggest that activating the LHA^OX^→SI circuit slows induction, whereas inhibition by Jaws has no accelerating effect.

We next analysed emergence from general anaesthesia in the same groups. RoRR was recorded as the emergence time. Mice were exposed to isoflurane 2 vol% for 30 min, after which isoflurane was shut off, and laser stimulation was initiated (Fig. 2k). The OxLight1 signals were completely suppressed during isoflurane. Two-way anova analysis of OxLight1 signals (*F*_(1, 10)_=21.82, *P*=0.0009) showed that laser stimulation led to an earlier increase in the OxLight1 signal during emergence (ChrimsonR laser *vs* ChrimsonR no-laser: 2 250 000 [282 000] *vs* 161 000 [315 000], uncorrected Fisher’s LSD, *P*=0.0001; *n*=6), but activation of Jaws did not prevent the increase in the OxLight1 signal during emergence (Jaws laser *vs* Jaws no-laser: 426 000 [126 000] *vs* 560 000 [170 000], uncorrected Fisher’s LSD, *P*=0.6985; *n*=6; Fig. 2k and l). Two-way anova of arousal behaviour data (*F*_(1, 10)_=29.64, *P*=0.0003) showed that the ChrimsonR group emerged faster with laser stimulation than the no-laser control (330 [16] *vs* 473 [15] s, uncorrected Fisher’s LSD, *P*<0.0001; *n*=6; Fig. 2m). In contrast, there were no significant changes in the Jaws group (Jaws laser *vs* Jaws no-laser: 429 [8] *vs* 434 [13] s, uncorrected Fisher’s LSD, *P*=0.7924; *n*=6; Fig. 2m). Comparisons between the ChrimsonR and Jaws groups showed no difference in the no-laser condition (uncorrected Fisher’s LSD, *P*=0.0556), but a significant difference in the laser condition (uncorrected Fisher’s LSD, *P*<0.0001; *n*=6; Fig. 2m). Overall, the results show that both induction and emergence were influenced by orexin concentrations in the SI.

### Selective stimulation of orexin terminals activates substantia innominata neurones and induces cortical activity

**Fig 3:**
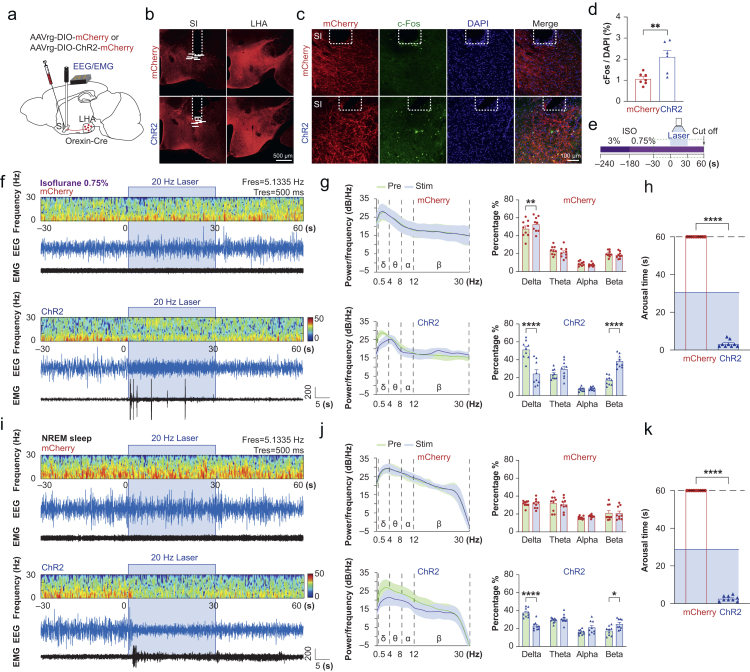
Selective activation of LHA^OX^→SI projection promotes arousal from anaesthesia sedation and NREM sleep. (a) Bilateral retrograde AAVrg-DIO-ChR2-mCherry or AAVrg-DIO-mCherry injection and optical fibre implantation into the SI, with EEG/EMG headpiece attached. (b) Expression of mCherry was detected in both SI and LHA. Each horizontal bar below represents the location of a fibre tip. (c) mCherry (red), cFos (green), and DAPI (blue) labelling in the SI after optogenetic stimulation. (d) Optogenetic activation increased cFos expression in the ChR2 group. Two-tailed unpaired *t*-test between two groups (*t*=3.507, df=11, *P*=0.0049). mCherry: *n*=7; ChR2: *n*=6. (e) Schematic of the arousal test under isoflurane 0.75 vol%. (f and i) EEG density spectral array (DSA), EEG, and EMG traces, before, during, and after optogenetic activation under isoflurane 0.75 vol% (f) or NREM sleep (i). (g and j) Comparison of EEG power spectral density (PSD) with 95% confidence bands and power percentage of EEG frequency bands between mCherry and ChR2 groups in 30 s phase before, and during laser stimulation under isoflurane 0.75 vol% (g), two-way anova with Šidák’s multiple comparisons. mCherry group: effect of laser stimulation: *F*_(1, 32)_=0.011, *P*=0.916, interaction between frequency and stimulation: *F*_(3, 32)_=6.621, *P*=0.0013; ChR2 group: effect of laser stimulation: *F*_(1, 32)_=0.007, *P*=0.932, interaction between frequency and stimulation: *F*_(3, 32)_=31.83, *P*<0.0001, or NREM sleep (j), mCherry group: effect of laser stimulation: *F*_(1, 32)_=0.007, *P*=0.935, interaction between laser stimulation and frequency: *F*_(3, 32)_=0.703, *P*=0.557; ChR2 group: effect of laser stimulation: *F*_(1, 32)_=0.007, *P*=0.932, interaction between stimulation and frequency: *F*_(3, 32)_=18.92, *P*<0.0001, *n*=9 for both groups. (h and k) Arousal times from stimulations during isoflurane 0.75 vol% (h), two-tailed unpaired *t*-test (*t*=90.87, df=16, *P*<0.0001), or NREM sleep (k), (*t*=121.6, df=16, *P*<0.0001), *n*=9 for both groups. ∗*P*≤0.05, ∗∗*P*≤0.01. anova, analysis of variance; LHA^OX^, lateral hypothalamic area orexinergic neurones; NREM, nonrapid eye movement; SI, substantia innominata.

Anterograde AAV injections into the LHA allowed us to stimulate terminals in the SI. However, this approach could potentially also activate fibres passing through the SI *en route* to other regions. To improve specificity, we injected the retrograde AAVrg-DIO-ChR2-mCherry or AAVrg-DIO-mCherry into the SI, implanted the optic fibre targeting the same region, and attached the EEG/EMG headmount (Fig. 3a). Robust ChR2-mCherry fluorescence was observed in both SI and LHA, confirming successful retrograde transport from orexin terminals in the SI to LHA cell bodies (Fig. 3b).

To confirm optogenetic activation of SI neurones, we used cFos staining, a marker of recent neuronal activity. Laser stimulation (470 nm, 20 Hz, 20 ms, 1 s on, 1 s off, 30 min) induced a significant increase in cFos staining in the SI compared with control animals expressing only mCherry (ChR2: 2.1 [0.3], *n*=6; mCherry: 1.1 [0.1], *n*=7; two-tailed unpaired *t*-test, *t*=3.507, df=11, *P*=0.0049; Fig. 3c and d), indicating that selective activation of orexin terminals effectively activated SI neurones.

We recorded EEG as a monitor of anaesthesia depth to determine the effect of LHA^OX^→SI circuit activation on cortical activity. We exposed mice expressing ChR2-mCherry or mCherry to isoflurane 0.75 vol% and performed arousal tests (Fig. 3e). Similar to the ChrimsonR activation, 473 nm stimulation of ChR2 (20 Hz, 20 ms, 30 s) reliably awakened the mice from isoflurane 0.75 vol% within 3.3 (0.6) s after stimulation onset (two-tailed unpaired *t*-test, *t*=90.87, df=16, *P*<0.0001, *n*=9; Fig. 3h). EEG PSD from the ChR2 group showed a transition to higher frequency and lower amplitude waveforms, marked by a decrease in delta (pre: 51.2 [3.4], stim: 24.3 [4.5]; Šidák’s multiple comparison test, pre *vs* stim, *P*<0.0001; *n*=9) and increases in beta (pre: 17.0 [1.7], stim: 38.4 [2.4]; Šidák’s multiple comparison test, pre *vs* stim, *P*<0.0001; *n*=9), consistent with arousal behaviour (two-way anova, *F*_(1, 32)_=0.007, *P*=0.932; Fig. 3f and g). EEG from the mCherry group showed a subtle increase in delta (pre: 47.7 [3.2], stim: 52.6 [2.8]; Šidák’s multiple comparison test, pre *vs* stim, *P*=0.0026; *n*=9) but no change in other brain waves (two-way anova, *F*_(1, 32)_=0.011, *P*=0.92; Fig. 3f and g).

### Selective activation of LHA^OX^→SI projection promotes emergence from nonrapid eye movement sleep

As anaesthesia and sleep share similar neural circuits, we examined whether the LHA^OX^→SI circuit regulates sleep/wake control. We stimulated freely moving animals with 470 nm laser pulses (20 Hz, 20 ms, 30 s) after they entered NREM sleep, as determined by EEG. Consistently, the EEG spectrum in the ChR2 group showed a decrease in the delta power (pre: 37.7 [1.7], stim: 23.0 [1.6], Šidák’s multiple comparison test, pre *vs* stim, *P*<0.0001; *n*=9) but an increase in beta (pre: 17.5 [1.7], stim: 25.0 [1.9]; Šidák’s multiple comparison test, pre *vs* stim, *P*=0.0105; *n*=9), indicating arousal from NREM sleep (two-way anova, *F*_(1, 32)_=0.007, *P*=0.93, Fig. 3i–k). In contrast, the mCherry control mice showed no response to optogenetic stimulation (two-way anova, *F*_(1, 32)_=0.007, *P*=0.935; *n*=9). Rapid arousal behaviour was observed from NREM sleep in the ChR2 group (2.7 [0.5] s) but not in the mCherry group (two-tailed unpaired *t*-test, *t*=121.6, df=16, *P*<0.0001, *n*=9, Fig. 3k). Thus activation of orexin terminals in the SI induces arousal from NREM sleep, similar to their role in promoting arousal from isoflurane sedation.

### Selective activation of LHA^OX^→SI projection induces analgesia

**Fig 4:**
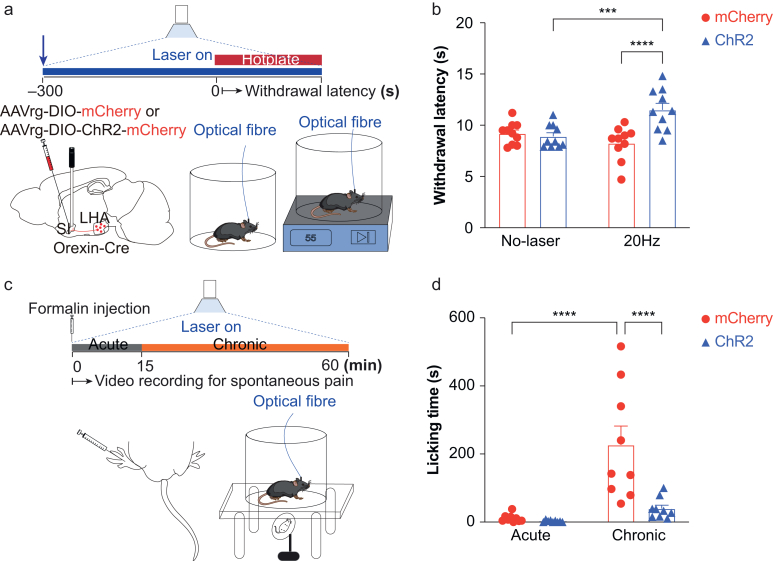
Selective activation of LHA^OX^→SI projection induces analgesia. (a) Retrograde AAVrg-DIO-ChR2-mCherry or AAVrg-DIO-mCherry was injected into the SI of Orexin-Cre mice bilaterally and optical fibres were implanted above. The mice were tested on a 55°C hotplate after 5 min of opto-stimulation with a 473 nm laser. (b) Optogenetic activation of LHA^OX^→SI increased the withdrawal latency to the thermal stimulation for the ChR2 laser group (20 Hz laser *vs* no-laser: 11.51 [0.64] *vs* 8.92 [0.36] s, uncorrected Fisher’s LSD, *P*=0.0002), but not the mCherry groups (20 Hz laser *vs* no-laser: 8.26 [0.52] *vs* 9.21 [0.34] s, uncorrected Fisher’s LSD, *P*=0.102). Two-way anova, the interaction between laser stimulation and groups *F*_(1, 18)_=20.61, *P*=0.0003; uncorrected Fisher’s LSD analysis of mCherry and ChR2 showed a significant difference in 20 Hz laser (*P*<0.0001), but not in no-laser (*P*=0.671); *n*=10 for both groups. (c) Formalin test. The left hind paws of the mice were injected with 10 μl of 5% formalin followed by a 60-min video recording with continuous laser stimulation. (d) Optogenetic activation of LHA^OX^→SI reduced the licking time for the ChR2 group during the chronic phase (mCherry *vs* ChR2: 226 [56] *vs* 39.67 [10] s, uncorrected Fisher’s LSD, *P*<0.0001), but not the acute phase (mCherry *vs* ChR2: 11.0 [3.9] *vs* 2.4 [0.9] s, uncorrected Fisher’s LSD, *P*=0.8323). Two-way anova, the interaction between laser stimulation and groups *F*_(1,__16__)_=9.383, *P*=0.0074, uncorrected Fisher’s LSD analysis of different phases showed a significant difference in the mCherry group (*P*<0.0001), but no difference in the ChR2 group (*P*=0.38`); *n*=9 for both groups. ∗∗∗*P*≤0.001, ∗∗∗∗*P*≤0.0001. All data are expressed as mean (sem). anova, analysis of variance; LHA^OX^, lateral hypothalamic area orexinergic neurones; LSD, least significant difference; sem, standard error of the mean; SI, substantia innominata.

As activating orexin neurones in LHA can induce analgesia,^40^ we determined whether SI is involved using the hotplate test to assess sensitivity to thermal pain. Mice expressing ChR2-mCherry or mCherry were placed on the hotplate after 5 min of continuous opto-stimulation (473 nm, 20 Hz, 20 ms, 1 s on, 1 s off), and withdrawal latency (time to paw withdrawal) was recorded (Fig. 4a). Two-way anova showed significance in the interaction of laser stimulation and group (*F*_(1, 18)_=20.61, *P*=0.0003). With laser stimulation, ChR2 mice showed a longer withdrawal latency (11.5 [0.6] s) than the same animals without laser stimulation (8.9 [0.4] s, uncorrected Fisher’s LSD, *P*=0.0002, *n*=10), and the mCherry group with laser stimulation (8.3 [0.5] s, uncorrected Fisher’s LSD, *P*<0.0001; *n*=10). No significant difference was observed in the mCherry and ChR2 group with no laser stimulation (uncorrected Fisher’s LSD, *P*=0.6712) nor in the mCherry group with or without laser stimulation (9.2 [0.3] s, uncorrected Fisher’s LSD, *P*=0.1021; Fig. 4b).

We also performed a formalin test to evaluate sensitivity to inflammatory pain. We injected 10 μl of 5% formalin s.c. into the dorsal part of the left hind paw followed by immediate laser stimulation, and pain behaviours (licking) were recorded for 1 h (Fig. 4c). The first 15-min phase, considered the acute phase, likely reflects direct nerve terminal stimulation, whereas the following 45 min, considered the chronic phase, reflects inflammatory pain responses. Two-way anova revealed a significant effect of group (*F*_(1,__16__)_=12.58, *P*=0.0027). Although no significant difference was observed during the acute phase (mCherry *vs* ChR2: 11.0 [3.9] *vs* 2.4 [0.9] s, uncorrected Fisher’s LSD, *P*=0.8323), activation of LHA^OX^→SI projection reduced licking time of the ChR2 mice during the chronic phase (mCherry *vs* ChR2: 226.56 [55.62] *vs* 39.67 [10.17], uncorrected Fisher’s LSD, *P*<0.0001; *n*=9; Fig. 4d). These results suggest that the LHA^OX^→SI projection is involved in analgesia for thermal and inflammatory pain.

### Orexinergic modulation of substantia innominata neurones in arousal from anaesthesia

**Fig 5:**
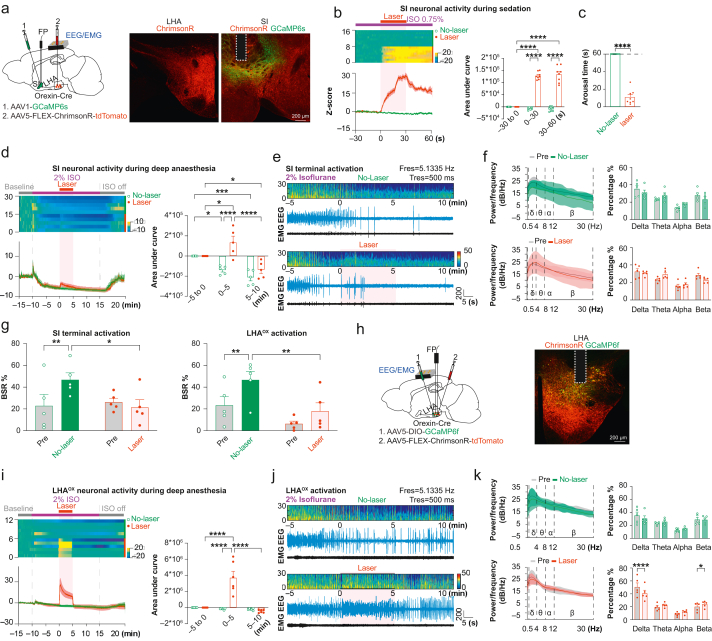
Optogenetic stimulation of LHA^OX^→SI regulates arousal at various depths of anaesthesia. (a) AAV5-FLEX-ChrimsonR-tdTomato injection into the LHA and AAV1-GCaMP6s injection into the SI, with optic fibres implanted and EEG/EMG headpieces attached. (b and d) GCaMP6s signals of SI neurones with or without optogenetic stimulation of orexin terminals in the SI during isoflurane 0.75 vol% sedation (b) or deep anaesthesia 2 vol% (d), and their AUC statistics. (b) Two-way anova, effect of time: *F*_(2, 28)_=95.36, *P*<0.0001, effect of stimulation: *F*_(1, 14)_=380.9, *P*<0.0001, the interaction: *F*_(2, 28)_=121.6, *P*<0.0001, Tukey’s multiple comparison test, *n*=8. (c) Arousal by laser stimulation while under isoflurane 0.75 vol%. (d) Two-way anova, effect of time: *F*_(2, 16)_=23.47, *P*<0.0001, effect of stimulation: *F*_(1, 8)_=12.73, *P*=0.0073, the interaction: *F*_(2, 16)_=11.77, *P*=0.0007, Tukey’s multiple comparison test, *n*=5. (e) EEG density spectral array (DSA), EEG and EMG traces with or without optogenetic stimulation in SI terminals under isoflurane 2 vol%. (f) EEG power spectral density (PSD) plots and frequency band analysis before, and during SI terminal stimulation. Two-way anova. No laser group: the interaction between stimulation and frequency: *F*_(3, 16)_=1.355, *P*=0.292, Šidák’s multiple comparisons, pre *vs* stim: delta: *P*=0.82, theta: *P*=0.815, alpha: *P*=0.751, beta: *P*=0.8; laser group: the interaction between stimulation and frequency: *F*_(3, 16)_=2.686, *P*=0.082, Šidák’s multiple comparisons, pre *vs* stim: delta: *P*=0.944, theta: *P*=0.228, alpha: *P*=0.963, beta: *P*=0.314; *n*=5. (g) Burst suppression ratio (BSR) of SI terminal activation and LHA^OX^ activation 5 min before and during stimulation. Two-way anova, uncorrected Fisher’s LSD, SI group: effect of the stimulation: *F*_(1, 8)_=1.345, *P*=0.2797, effect of the time: *F*_(1, 8)_=5.605, *P*=0.0454, interaction between stimulation and time: *F*_(1, 8)_=12.8, *P*=0.007; LHA^OX^ group: effect of the stimulation: *F*_(1, 8)_=6.984, *P*=0.0296, effect of the time: *F*_(1, 8)_=14.67, *P*=0.005, interaction between stimulation and time: *F*_(1, 8)_=1.65, *P*=0.235; *n*=5 for each group. (h) AAV5-FLEX-ChrimsonR-tdTomato and AAV5-DIO-GCaMP6f injections into the LHA, with fibre and EEG/EMG implantations. (i) GCaMP6f signals of LHA^OX^ neurones during deep anaesthesia 2 vol%. Two-way anova, effect of time: *F*_(2, 20)_=22.85, *P*<0.0001, effect of stimulation: *F*_(1, 10)_=22.92, *P*=0.0007, the interaction: *F*_(2, 20)_=23.36, *P*<0.0001, Tukey’s multiple comparison test, *n*=6. (j) EEG DSA, EEG and EMG traces with or without optogenetic stimulation in the LHA under isoflurane 2 vol%. (k) EEG PSD plots and frequency band analysis before and during LHA stimulation. Two-way anova. No laser group: the interaction between stimulation and frequency: *F*_(3, 16)_=1.175, *P*=0.3503, Šidák’s multiple comparisons, pre *vs* stim: delta: *P*=0.4969, theta: *P*=0.8968, alpha: *P*=0.8884, beta: *P*>0.9999; laser group: the interaction between stimulation and frequency: *F*_(3, 16)_=24.35, *P*<0.0001, Šidák’s multiple comparisons, pre *vs* stim: delta: *P*<0.0001, theta: *P*=0.1235, alpha: *P*=0.0781, beta: *P*=0.0492; *n*=5. ∗*P*≤0.05, ∗∗*P*≤0.01, ∗∗∗*P*≤0.001, ∗∗∗∗*P*≤0.0001. All data are expressed as mean (sem). anova, analysis of variance; AUC, area under the curve; FP, fibre photometry; LHA^OX^, lateral hypothalamic area orexinergic neurones; LSD, least significant difference; sem, standard error of the mean; SI, substantia innominata.

To examine the role of SI neurones in arousal from varying depths of general anaesthesia, we used a photometry-optogenetic approach, stimulating LHA^OX^→SI projections while recording SI activity through a single fibre (Fig. 5a). Under light anaesthesia (isoflurane 0.75 vol%), optogenetic stimulation of orexin terminals elicited a rapid increase in GCaMP6s fluorescence owing to Ca^2+^ influx (Fig. 5b). Area under the curve was greater in the laser stimulated group than in the control group during stimulation (128 000 [5360] *vs* –6920 [1840], Tukey’s multiple comparisons, *P*<0.0001) and after stimulation (134 000 [11 500] *vs* –8900 [3120]; Tukey’s multiple comparisons, *P*<0.0001), indicating that SI neurones receive excitatory input from orexin neurones and are highly responsive under light anaesthesia (two-way anova, *F*_(2, 28)_=121.6, *P*<0.0001; *n*=8). This response correlated with robust arousal behaviour (11.0 [2.9] s *vs* no arousal within 60 s, two-tailed unpaired *t*-test, *t*=16.67, df=14, *P*<0.0001; *n*=8; Fig. 5c).

For deep anaesthesia (isoflurane 2 vol%), Ca^2+^ signals declined steadily, reflecting deepening anaesthesia (no laser group: –5 to 0 min: –0.4 [1.5]; 0–5 min: –140 000 [18 700]; 5–10 min: –213 000 [33 700]; Tukey’s multiple comparisons, –5 to 0 *vs* 0–5 min, *P*=0.0102, –5 to 0 *vs* 5–10 min, *P*=0.0003). Activation of the LHA^OX^→SI projection produced a small increase in GCaMP6s signal (laser group: –5 to 0 min: –6.8 [1.4]; 0–5 min: 135 000 [61 600]; 5–10 min: –137 000 [37 200]; Tukey’s multiple comparisons, laser group: –5 to 0 *vs* 5–10 min, *P*=0.0116; no laser 0–5 *vs* laser 0–5 min, *P*<0.0001, laser –5 to 0 *vs* laser 0–5 min, *P*=0.0127, laser 0–5 *vs* laser 5–10 min, *P*<0.0001; two-way anova, *F*_(2, 16)_=11.77, *P*=0.0007; *n*=5; Fig. 5d), yet no observable arousal behaviours or changes in EEG PSD power (two-way anova, *F*_(1, 16)_=0.0022, *P*=0.9631 for no laser group; *F*_(1, 16)_=0.0037, *P*=0.952 for laser group; Šidák’s multiple comparison test; *n*=5; Fig. 5e and f).

To directly stimulate orexinergic cell bodies in LHA during deep anaesthesia, Cre-dependent ChrimsonR and GCaMP6f were injected into LHA (Fig. 5h). GCaMP6f signals from orexin neurons increased upon stimulation (no laser group: –5 to 0 min: –0.4 [1.9], 0–5 min: –151 000 [37 800]; 5–10 min: –220 000 [41 800]; laser group: –5 to 0 min: –20.2 [8.9], 0–5 min: 3 710 000 [797 000], 5–10 min: –446 000 [81 500]; Tukey’s multiple comparisons, no laser 0–5 *vs* laser 0–5 min, *P*<0.0001; laser –5 to 0 *vs* laser 0–5 min, *P*<0.0001; laser 0–5 *vs* laser 5–10 min, *P*<0.0001; two-way anova, *F*_(2, 20)_=23.36, *P*<0.0001; *n*=6, Fig. 5i). In contrast to SI, stimulation of LHA^OX^ induced pronounced EEG PSD changes, including a decrease in delta power (two-way anova, *F*_(1, 16)_=0.0506, *P*=0.8249 for laser group; Šidák’s multiple comparison test; pre: 50.6 [6.4] *vs* laser: 40.6 [4.9], *P*<0.0001) and increase in beta power (pre: 20.0 [3.0] *vs* laser: 23.9 [2.2], *P*=0.049; *n*=5), indicative of broader brain activation (Fig. 5j and k), but with no arousal behaviour observed.

**Fig 6:**
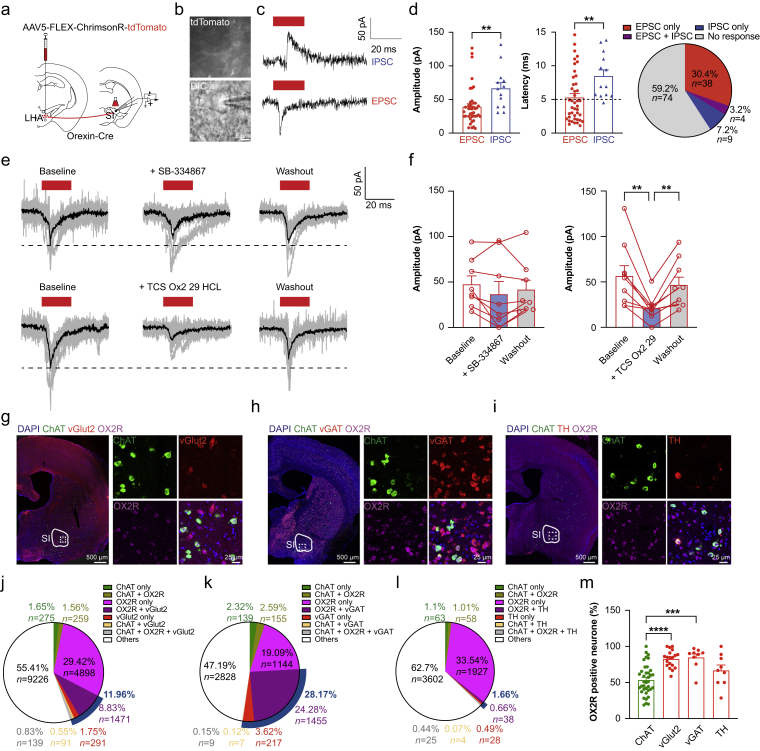
Substantia innominata (SI) neurones receive monosynaptic excitatory inputs from LHA^OX^. (a) AAV5-FLEX-ChrimsonR-tdTomato was injected into the LHA of Orexin-Cre mice, and patch-clamp whole-cell recordings were performed on the SI neurones. (b) Abundant tdTomato-positive fibres surrounding SI neurones. (c) Both excitatory postsynaptic currents (EPSCs) and inhibitory postsynaptic currents (IPSCs) were elicited by photostimulation (635 nm). (d) Amplitude (EPSC: 39.4 [4.3] pA, *n*=42; IPSC: 66.6 [8.5] pA, *n*=13; two-tailed unpaired *t*-test, *t*=3.012, df=53, *P*=0.004), latency (EPSC: 5.3 [0.6] ms, *n*=42; IPSC: 8.5 [0.9] ms, *n*=13; two-tailed unpaired *t*-test, *t*=2.785, df=53, *P*=0.0074), and distribution. (e and f) Laser-evoked EPSCs were blocked by the OX2R antagonist TCS Ox2 29, but not as much by the OX1R antagonist SB-334867. One-way anova, effect of SB-334867, *F*_(1.536, 10.75)_=1.944, *P*=0.1928, Tukey’s multiple comparisons of the amplitude of the baseline (47.7 [9.4] pA), SB-334867 (36.8 [14.1] pA), and washout (41.9 [10.7] pA) showed no significant difference (baseline *vs* SB-334867: *P*=0.2712, SB-334867 *vs* washout: *P*=0.6891); *n*=8. One-way anova analysis of effect of the TCS OX2 29, *F*_(1.262, 10.09)_=17.38, *P*=0.0012, Tukey’s multiple comparisons of baseline (56.8 [11.7] pA), TCS OX2 29 (21.1 [4.5] pA), and washout (46.9 [8.7] pA) showed TCS OX2 29 significantly decreased the amplitude of the laser evoked EPSCs (baseline *vs* TCS OX2 29: *P*=0.006, TCS OX2 *vs* washout: *P*=0.0058); *n*=9. *N* stands for cell number, each data point represents an average of five traces from a single cell. (g–i) RNAscope fluorescence for different types of neurones (ChAT, vGlut2, vGAT, TH positive) and labelling with OX2R in SI. (j–l) Colocalisations of ChAT, OX2R, and vGlut2 (j); or vGAT (k), or TH (l). (m) OX2R distributions in ChAT, vGlut2, vGAT, and TH neurones in SI. One-way anova, *F*_(3, 70)_=13.66, *P*<0.0001, Tukey’s multiple comparisons test for OX2R+ChAT/ChAT (53.6 [3.5]%), OX2R+vGlut2/vGlut2 (82.6 [2.7]%), OX2R+vGAT/vGAT (84.8 [4.9]%), OX2R+TH/TH (67.0 [7.4]%) showed OX2R are distributed more in vGlut2 (*P*<0.0001) and vGAT (*P*=0.0001) than ChAT neurones. ChAT neurones were counted from 37 brain slices of five mice, vGlut2 neurones from 19 brain slices of five mice, and vGAT and TH neurones from 9 brain slices of five mice. ∗∗*P*≤0.01, ∗∗∗*P*≤0.001, ∗∗∗∗*P*<0.0001. All data are expressed as mean (sem). anova, analysis of variance; FP, fibre photometry; LHA^OX^, lateral hypothalamic area orexinergic neurones; OX1R, orexin receptor-1; OX2R, orexin receptor-2; sem, standard error of the mean.

The BSR, a metric for assessing deep anaesthesia, increased with declining GCaMP signals in both SI groups (two-way anova, *F*_(1, 8)_=12.8, *P*=0.0072; uncorrected Fisher’s LSD; no laser group: pre: 22.9 [10.5] *vs* no laser: 46.8 [6.4], p = 0.003; laser group: pre: 26.3 [3.5] *vs* laser: 21.4 [7.0], *P*=0.417; Fig. 5g, left) and LHA group (two-way anova, *F*_(1, 8)_=1.65, *P*=0.235; uncorrected Fisher’s LSD; no laser group: pre: 23.4 [8.0] *vs* no laser: 46.7 [8.0], *P*=0.0068; laser group: pre: 6.3 [2.4] *vs* laser: 17.9 [7.7], *P*=0.1096; Fig. 5g, right). However, laser stimulation mitigated this BSR increase in both groups (no laser *vs* laser: *P*=0.025; *n*=5 in SI; *P*=0.0097; *n*=5 in LHA). These data suggest that SI activation via orexin terminals modified BSR but not EEG PSD, whereas LHA^OX^ activation altered BSR and EEG PSD, but neither overcame the profound suppression of deep anaesthesia to elicit behavioural arousal.

### Excitatory and inhibitory postsynaptic currents are elicited by stimulating orexin terminals in the substantia innominata

To study synaptic transmission in the SI, AAV5-FLEX-ChrimsonR-tdTomato was injected into the LHA, and brain slices containing the SI region were used for whole-cell patch-clamp electrophysiology recordings (Fig. 6a and b). Upon applying a short train of the red laser pulses (635 nm, 20 Hz, 20 ms), both EPSCs and IPSCs were detected in SI neurones (Fig. 6c). Among all the tested neurones, 40.8% responded to laser stimulation. Most responses were EPSCs (30.4%, *n*=38), with fewer IPSCs (7.2%, *n*=9) and both EPSCs and IPSCs (3.2%, *n*=4). The amplitudes of EPSCs (39.4 [4.3] pA, *n*=42) were lower than those of IPSCs (66.6 [8.5] pA, *n*=13; two-tailed unpaired *t*-test, *t*=3.012, df = 53, *P*=0.004). The EPSCs had shorter latencies of ∼5 ms (5.3 [0.6] ms, *n*=42) indicating that they were monosynaptic connections.^41^ However, IPSCs had a significantly delayed response (8.5 [0.9] ms, *n*=13, two-tailed unpaired *t*-test, *t*=2.785, df=53, *P*=0.0074; Fig. 6d), suggesting indirect innervation by orexin neurones. EPSCs were reversibly blocked by the OX2R antagonist TCS-Ox2-29 (one-way anova, *F*_(1.262, 10.09)_=17.38, *P*=0.0012, Tukey’s multiple comparisons, baseline *vs* TCS OX2 29: *P*=0.006, TCS OX2 *vs* washout: *P*=0.0058; *n*=9), but not by the OX1R antagonist SB-334867 (one-way anova, *F*_(1.536, 10.75)_=1.944, *P*=0.1928, Tukey’s multiple comparisons, baseline *vs* SB-334867: *P*=0.2712, SB-334867 *vs* washout: *P*=0.6891; *n*=8; Fig. 6e and f). IPSCs exhibited similar responses to the blockers (SB-334867: one-way anova, *F*_(1.317, 9.218)_=5.014, *P*=0.044, Tukey’s multiple comparisons, baseline *vs* SB-334867: *P*=0.103, SB-334867 *vs* washout: *P*=0.178; *n*=8; TCS-Ox2-29: one-way anova, *F*_(1.14, 7.981)_=11.42, *P*=0.0085, Tukey’s multiple comparisons, baseline *vs* TCS OX2 29: *P*=0.0145, TCS OX2 *vs* washout: *P*=0.047; *n*=8; Supplementary Fig. 2c and d). This indicated that OX2Rs but not OX1Rs in the SI are involved in both excitatory and inhibitory synaptic transmission.

### Downstream neurones of the lateral hypothalamic area orexinergic neurones in the substantia innominata

We further investigated the downstream neuronal types in SI that are innervated by orexin terminals. Using RNAscope, four neuronal types were examined by *in situ* hybridisation for colocalisation with OX2R mRNA: ChAT (cholinergic), Slc17a6 (vGlut2, glutamatergic), Slc32a1 (vGAT, GABAergic), and TH (catecholaminergic). Although SI has been known as the major source of cholinergic neurones, only a small portion of SI cells were cholinergic neurones (∼4.1% of all SI cells, average of ChAT staining from Fig. 6g–i). There were significantly more glutamatergic neurones (12.0%; Fig. 6g and j) and GABAergic neurones (28.2%; Fig. 6h and k), along with a smaller population number of catecholaminergic neurones (1.7%; Fig. 6i and l). Because of the lack of ability to co-stain four probes at once, we used the ChAT probe as a reference in all sets of RNAscope staining to compare the ratios between different neuronal types. The analysis revealed ratios of about 2.8 for vGlut2/ChAT, 8.9 for vGAT/ChAT, and 0.7 for TH/ChAT (Supplementary Fig. 3a and b). We also calculated the percentage of OX2R colocalisation for each neuronal type. For ChAT neurones, 53.6% were double-positive for OX2R. Similarly, 82.6% of vGlut2 neurones, 84.8% of vGAT neurons, and 67.0% of TH cells co-expressed OX2R (Fig. 6m). Among the OX2R^+^ neurones, 22.3% were double-positive for vGlut2, 51.4% for vGAT, 2.5% for TH, and 4.5% for ChAT (Supplementary Fig. 3c and d). Taken together, these data indicate that all four neuronal types in the SI express OX2R, with vGAT positive neurones being the most abundant and showing the highest level of OX2R expression.

## Discussion

We investigated the LHA^OX^→SI circuit in Orexin-Cre mice using optogenetics, *in vivo* fibre photometry, EEG, EMG, RNAscope, and electrophysiology. We show that activation of LHA^OX^→SI projections arouses animals from both NREM sleep and isoflurane sedation, delays induction of isoflurane anaesthesia, accelerates recovery from anaesthesia, and enhances tolerance to thermal pain and formalin-induced inflammatory pain. These effects are primarily mediated by OX2Rs, which are expressed on cholinergic, glutamatergic, and GABAergic neurones in SI.

In clinical practice, anaesthetic concentrations are adjusted based on the pain intensity of diagnostic or therapeutic procedures. In rodents, the minimal alveolar concentration of isoflurane varies with factors such as age, strain, and physiological state.^42^ In this study, three different concentrations of isoflurane were used to simulate various clinical scenarios. To model sedation for less invasive procedures, we used isoflurane 0.75 vol%. For induction, a brief isoflurane 3 vol% was selected, reflecting clinical practice where induction doses exceed maintenance concentrations to ensure rapid onset. Finally, isoflurane 2 vol% was applied to maintain surgical anaesthesia.

Under isoflurane 0.75 vol% sedation, activation of the LHA^OX^→SI projection elicited robust arousal behaviours alongside changes in EEG and EMG signals. At isoflurane 2 vol%, neither LHA^OX^→SI activation nor LHA^OX^ stimulation induced arousal behaviours. However, LHA^OX^→SI activation reduced EEG BSR without affecting PSD, whereas LHA^OX^ stimulation altered both BSR and PSD. BSR reflects electrical suppression of the brain (time-based suppression), whereas PSD reflects brain activation (frequency-based rhythms). These results suggest that under deep anaesthesia, SI activation generates a focal effect insufficient to propagate cortically, whereas LHA^OX^ activation engages broader neural networks as evidenced by the PSD shifts.

The brain arousal system consists of multiple neural pathways that safeguard survival. The orexin circuit is a key modulator in arousal control through its extensive projections. The SI is part of the basal forebrain (BF), located between the amygdala and the globus pallidus, and is known as the major source of cholinergic inputs to the cerebral cortex, relaying ascending arousal projections from the brainstem to the cortex.^43^ Previous studies with different approaches have demonstrated the role of orexin signalling on various targets within the BF. For example, microinjection of orexin A into the BF accelerates emergence from anaesthesia, whereas blocking OX1Rs delays recovery.^44^ Similarly, intrabasalis microinjections of orexins enhance cortical acetylcholine release and EEG activation via OX1R during anaesthesia.^45^ Optogenetic activation of orexin terminals in BF and LC also shortens recovery time and reduces burst suppression under isoflurane.^46^ Other studies highlight that orexin A but not co-released dynorphine excites cholinergic neurones in the magnocellular preoptic nucleus (MCPO) to promote wakefulness.^47^ Moreover, orexin receptor antagonism enhances sleep-related adenosine and GABA release in the BF, revealing additional layers of modulation within this system.^48^

Other arousal-related systems include adrenergic neurones in the LC, histaminergic neurones in the tuberomammillary nucleus, serotoninergic neurones in the raphe nuclei, and dopaminergic neurones in the VTA, all of which have intricate connections with orexin neurones.^49^ These nuclei collectively influence anaesthesia-induced arousal control.^4^^,^^19^^,^^45^^,^^50^ When one arousal system is not working properly, other systems can compensate to maintain the appropriate wakefulness. This might explain why anaesthesia induction and emergence speed were not significantly altered when Jaws was activated in the SI. Similarly, induction speed was unaffected by orexin neurone ablation.^50^ On the contrary, when the orexin arousal system was activated, the system arousal level was elevated, therefore induction of anaesthesia was delayed, emergence was accelerated, and reanimation can be triggered during light anaesthesia. In contrast to optogenetic stimulation, previous studies using orexin ligand microinjection were not able to show an effect on induction time.^44^^,^^51^ The dynamics of OxLight1 signals further supported the association between increased orexin release and changes in arousal during anaesthesia.

About half of SI cells expressed orexin receptors as shown by *in situ* hybridisation, with optogenetic stimulation of orexin terminals eliciting mostly EPSCs (30.4%) and some IPSCs (7.2%). The short latency and good signal-to-noise ratio of EPSCs indicate direct monosynaptic connections, with OX2R, but not OX1R, receptors involved. The longer latency of IPSCs suggests indirect connections through inhibitory interneurones. Previous studies have shown that orexin neurones co-release glutamate and dynorphin in addition to orexins, which might explain why the EPSCs cannot be fully blocked by an OX2R antagonist. Previous pharmacological studies have reported diverse distributions of orexin receptors.^52^ Our data show that OX2R is the dominant receptor in the SI, with 96% of orexin receptor-positive cells expressing OX2R. A survey of other sites in our brain slices showed that the LC primarily contains OX1R, whereas the AHN and PVT express both OX1R and OX2R (Fig. 1c, Supplementary Fig. 1). It is well known that LC is the main source of norepinephrine in the brain, and SI is the main source of acetylcholine. Although both are important in arousal regulation, likely influencing different aspects of arousal, they achieve their functions through different orexin receptors. The OX1R in the LC is involved in fear memory consolidation^53^ and reward-seeking behaviour.^54^ Such functional differences between OX1R and OX2R merit further investigation.

The SI contains a diverse population of neurones, including cholinergic, GABAergic, and glutamatergic neurones. GABAergic neurones have complex heterogeneous subgroups that exhibit tonic or phasic activity, which could be associated with gating activity in cortical neurones. About 40% of GABAergic neurones in the BF increased their discharge rate with somatosensory evoked cortical activation, whereas the rest were suppressed.^55^ The BF GABAergic neurones are typically smaller than cholinergic neurones, and function not only as inhibitory interneurones to regulate nearby neurones but also as projecting neurones to the neocortex.^56^ Somatostatin^+^ GABAergic neurones promote sleep, whereas parvalbumin^+^ GABAergic neurones and glutamatergic neurones promote arousal.^57^ The fast-spiking parvalbumin^+^ neurones have been recognised as important players in generating gamma oscillations.^58^ Future studies should focus on selectively activating these different types of neurones in the SI to further explore their roles in arousal and analgesia.

Orexin activation, through chemogenetics or optogenetics, has been shown to modulate pain perception.^20^^,^^26^^,^^59^ The orexin system targets various brain regions involved in pain processing, including the periaqueductal grey,^60^^,^^61^ rostral ventromedial medulla,^24^ medial preoptic area,^62^ and VTA.^63^ Here, we show that the SI is also involved in pain control. The pain pathway involves peripheral reception, spinal cord ascending transmission, and brain interpretation. Orexinergic neurones project widely throughout the brain and spinal cord, suggesting that multiple sites are involved in processing pain signals. More research is needed to reveal the mechanisms by which the LHA^OX^→SI pathway regulates pain.

## Authors' contributions

Designed and performed research: WZ, XX

Electrophysiology experimental design: FW, WZ

Data analysis: FW, XX

Virus production and RNAscope design: CC, XX

Scientific adviser: ZG

Project administration and funding acquisition: WZ

Wrote the manuscript: WZ, XX

Substantial contributions to the research, read and approved the submitted manuscript, and agreed to be responsible for the content: all authors

## Data availability statement

All data generated in this study are included within the main text or supplementary materials, with source data provided. This includes individual data points and averaged values presented in both the figures and supplementary information. The corresponding authors can provide raw data from optogenetic stimulation, *in vivo* fibre photometry, *in vitro* electrophysiology, and other experiments upon request.

## Funding

US National Institutes of Health (K08GM138981 to WZ); University of California, San Francisco, Department of Anesthesia.

## Declaration of interest

The authors declare no competing interests.

## Figures and Tables

**Figure:**
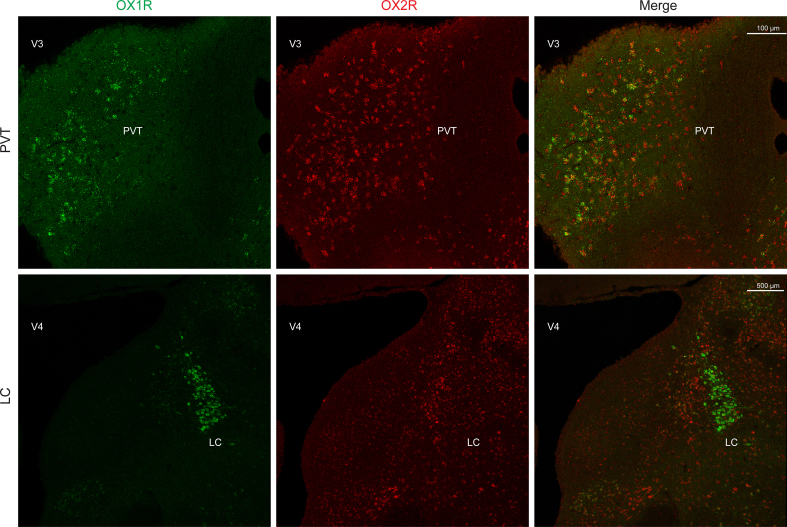
**OX1R and OX2R distributions at different regions. RNAscope staining of OX1R and OX2R at the paraventricular nucleus of the Thalamus (PVT) and the locus coeruleus (LC) shows the predominant OX1R staining in the LC but evenly distributed OX1R and OX2R in the PVT**.

**Figure:**
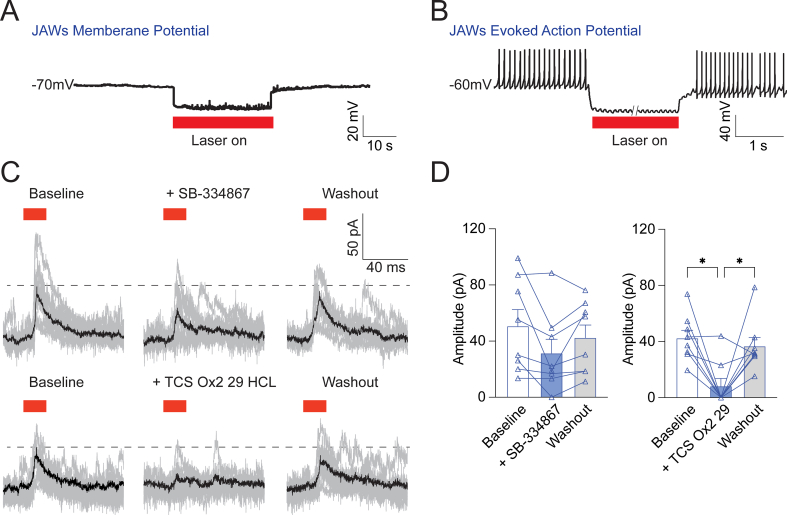
**Acute brain slice electrophysiology recordings. A, B. AAV5-FLEX-JAWs-GFP was injected into the LHA of Orexin-Cre mice. Patch-clamp whole-cell recordings on LHA orexin neurons showed optogenetic activation of JAWs decreased the membrane potential (A) and silenced the action potentials evoked by injecting 50 pA current at 10 Hz (B). C. The IPSCs can be blocked by the OX2R antagonist - TCS Ox2 29, but not by the OX1R antagonist - SB-334867. D. The amplitudes before, during, and after blockage. One-way ANOVA, effect of the SB-334867, F(1.317, 9.218) = 5.014, p = 0.0441, Tukey’s multiple comparisons of the baseline (50.88 ± 11.69 pA), SB-334867 (31.52 ± 9.9 pA), and washout (42.54 ± 9.026 pA) showed no significant difference (baseline vs SB-334867: p = 0.1028, SB-334867 vs washout: p = 0.1778); n = 8. One-way ANOVA, effect of the TCS OX2, F(1.14, 7.981) = 11.42, p = 0.0085, Tukey’s multiple comparisons of the baseline (42.58 ± 5.9 pA), TCS OX2 (8.39 ± 5.83 pA), and washout (36.85 ± 6.55 pA) showed TCS OX2 29 significantly decreased the amplitude of the laser evoked IPSCs (baseline vs TCS OX2 29: p = 0.0145, TCS OX2 vs washout: p = 0.047); n = 8. n stands for cell numbers, each data point represents the average of five traces from a single cell. ∗p ≤ 0.05. ∗p ≤ 0.05. All data are expressed as mean ± S.E.M**.

**Figure:**
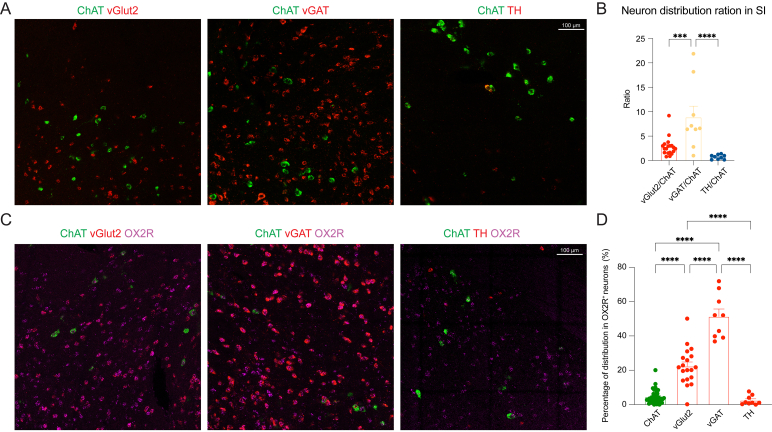
**Colocalization of OX2R, ChAT, vGlut2, vGAT, and TH in the SI. A. Double-staining of vGlut2/ChAT, vGAT/ChAT, and TH/ChAT. B. Ratios of vGlut2/ChAT, vGAT/ChAT, and TH/ChAT. Ordinary one-way ANOVA, F(2, 34) = 12.9, p < 0.0001), Tukey multiple comparisons showed the ratio of vGAT/ChAT (8.86 ± 2.28, n = 9 brain slices from 5 mice) was significantly higher than vGlut2/ChAT (2.81 ± 0.43, p = 0.0006, n = 19 brain slices from 5 mice) and TH/ChAT (0.73 ± 0.16, p < 0.0001, n = 9 brain slices from 5 mice). C. The co-localization of ChAT, vGlut2, vGAT, TH, and OX2R. D. The percentage of ChAT, vGlut2, vGAT, and TH neurons in the OX2R+ neurons. Data was calculated from the number of double positive neurons divided by the total number of OX2R+ neurons. Ordinary one-way ANOVA, F(3, 70) = 105.2, p < 0.0001. Tukey’s multiple comparisons showed vGlut2 (22.34 ± 2.48 %, n = 19 brain slices from 5 mice), vGAT (51.38 ± 4.31 %, n = 9 brain slices from 5 mice) ChAT (4.53 ± 0.66 %, n = 37 brain slices from 5 mice), and TH (2.47 ± 0.9 %, n = 9 brain slices from 5 mice) are different from each other. ∗∗∗∗p < 0.0001. All data are expressed as mean ± S.E.M**.

## References

[1] Steen P.A., Michenfelder J.D. (1979). Neurotoxicity of anesthetics. Anesthesiology.

[2] Neukirchen M., Kienbaum P. (2008). Sympathetic nervous system: evaluation and importance for clinical general anesthesia. Anesthesiology.

[3] Mashour G.A., Palanca B.J., Basner M. (2021). Recovery of consciousness and cognition after general anesthesia in humans. Elife.

[4] Moody O.A., Zhang E.R., Vincent K.F. (2021). The neural circuits underlying general anesthesia and sleep. Anesth Analg.

[5] Sakurai T., Amemiya A., Ishii M. (1998). Orexins and orexin receptors: a family of hypothalamic neuropeptides and G protein-coupled receptors that regulate feeding behavior. Cell.

[6] de Lecea L., Kilduff T.S., Peyron C. (1998). The hypocretins: hypothalamus-specific peptides with neuroexcitatory activity. Proc Natl Acad Sci U S A.

[7] Tyree S.M., Jennings K.J., Gonzalez O.C. (2023). Optogenetic and pharmacological interventions link hypocretin neurons to impulsivity in mice. Commun Biol.

[8] Inutsuka A., Inui A., Tabuchi S., Tsunematsu T., Lazarus M., Yamanaka A. (2014). Concurrent and robust regulation of feeding behaviors and metabolism by orexin neurons. Neuropharmacology.

[9] Elam H.B., Perez S.M., Donegan J.J., Lodge D.J. (2021). Orexin receptor antagonists reverse aberrant dopamine neuron activity and related behaviors in a rodent model of stress-induced psychosis. Transl Psychiatry.

[10] Soya S., Shoji H., Hasegawa E. (2013). Orexin receptor-1 in the locus coeruleus plays an important role in cue-dependent fear memory consolidation. J Neurosci.

[11] Lin L., Faraco J., Li R. (1999). The sleep disorder canine narcolepsy is caused by a mutation in the hypocretin (orexin) receptor 2 gene. Cell.

[12] Ito H., Fukatsu N., Rahaman S.M. (2023). Deficiency of orexin signaling during sleep is involved in abnormal REM sleep architecture in narcolepsy. Proc Natl Acad Sci U S A.

[13] Chemelli R.M., Willie J.T., Sinton C.M. (1999). Narcolepsy in orexin knockout mice: molecular genetics of sleep regulation. Cell.

[14] Flanigan M.E., Aleyasin H., Li L. (2020). Orexin signaling in GABAergic lateral habenula neurons modulates aggressive behavior in male mice. Nat Neurosci.

[15] Tung L.W., Lu G.L., Lee Y.H. (2016). Orexins contribute to restraint stress-induced cocaine relapse by endocannabinoid-mediated disinhibition of dopaminergic neurons. Nat Commun.

[16] Date Y., Ueta Y., Yamashita H. (1999). Orexins, orexigenic hypothalamic peptides, interact with autonomic, neuroendocrine and neuroregulatory systems. Proc Natl Acad Sci U S A.

[17] Bulbul M., Babygirija R., Ludwig K., Takahashi T. (2010). Central orexin-A increases gastric motility in rats. Peptides.

[18] Varga A.G., Whitaker-Fornek J.R., Maletz S.N., Levitt E.S. (2022). Activation of orexin-2 receptors in the Kӧlliker-Fuse nucleus of anesthetized mice leads to transient slowing of respiratory rate. Front Physiol.

[19] Li J., Li H., Wang D. (2019). Orexin activated emergence from isoflurane anaesthesia involves excitation of ventral tegmental area dopaminergic neurones in rats. Br J Anaesth.

[20] Inutsuka A., Yamashita A., Chowdhury S. (2016). The integrative role of orexin/hypocretin neurons in nociceptive perception and analgesic regulation. Sci Rep.

[21] Suzuki M., Shiraishi E., Cronican J., Kimura H. (2024). Effects of the orexin receptor 2 agonist danavorexton on emergence from general anaesthesia and opioid-induced sedation, respiratory depression, and analgesia in rats and monkeys. Br J Anaesth.

[22] Yan J.A., Ge L., Huang W., Song B., Chen X.W., Yu Z.P. (2008). Orexin affects dorsal root ganglion neurons: a mechanism for regulating the spinal nociceptive processing. Physiol Res.

[23] van den Pol A.N. (1999). Hypothalamic hypocretin (orexin): robust innervation of the spinal cord. J Neurosci.

[24] Azhdari-Zarmehri H., Semnanian S., Fathollahi Y. (2014). Orexin-A microinjection into the rostral ventromedial medulla causes antinociception on formalin test. Pharmacol Biochem Behav.

[25] Shakerinava P., Sayarnezhad A., Karimi-Haghighi S., Mesgar S., Haghparast A. (2022). Antagonism of the orexin receptors in the ventral tegmental area diminished the stress-induced analgesia in persistent inflammatory pain. Neuropeptides.

[26] Kaneko T., Oura A., Imai Y. (2024). Orexin neurons play contrasting roles in itch and pain neural processing via projecting to the periaqueductal gray. Commun Biol.

[27] Xiang X., Chen Y., Li K.X. (2022). Neuroanatomical basis for the orexinergic modulation of anesthesia arousal and pain control. Front Cell Neurosci.

[28] Scharf M.T., Kelz M.B. (2013). Sleep and anesthesia interactions: a pharmacological appraisal. Curr Anesth Rep.

[29] Ozen Irmak S., de Lecea L. (2014). Basal forebrain cholinergic modulation of sleep transitions. Sleep.

[30] Zhu Z., Ma Q., Miao L. (2021). A substantia innominata-midbrain circuit controls a general aggressive response. Neuron.

[31] Jones B.E. (2004). Activity, modulation and role of basal forebrain cholinergic neurons innervating the cerebral cortex. Prog Brain Res.

[32] Leung L.S., Chu L., Prado M.A.M., Prado V.F. (2021). Forebrain acetylcholine modulates isoflurane and ketamine anesthesia in adult mice. Anesthesiology.

[33] Xu M., Chung S., Zhang S. (2015). Basal forebrain circuit for sleep-wake control. Nat Neurosci.

[34] Cui Y., Lv G., Jin S. (2017). A central amygdala-substantia innominata neural circuitry encodes aversive reinforcement signals. Cell Rep.

[35] Dafny N., Dong W.Q., Prieto-Gomez C., Reyes-Vazquez C., Stanford J., Qiao J.T. (1996). Lateral hypothalamus: site involved in pain modulation. Neuroscience.

[36] Wang D., Pan X., Zhou Y. (2023). Lateral septum-lateral hypothalamus circuit dysfunction in comorbid pain and anxiety. Mol Psychiatry.

[37] Duffet L., Kosar S., Panniello M. (2022). A genetically encoded sensor for in vivo imaging of orexin neuropeptides. Nat Methods.

[38] Klapoetke N.C., Murata Y., Kim S.S. (2014). Independent optical excitation of distinct neural populations. Nat Methods.

[39] Chuong A.S., Miri M.L., Busskamp V. (2014). Noninvasive optical inhibition with a red-shifted microbial rhodopsin. Nat Neurosci.

[40] Bingham S., Davey P., Babbs A. (2001). Orexin-A, an hypothalamic peptide with analgesic properties. Pain.

[41] Holloway B.B., Stornetta R.L., Bochorishvili G., Erisir A., Viar K.E., Guyenet P.G. (2013). Monosynaptic glutamatergic activation of locus coeruleus and other lower brainstem noradrenergic neurons by the C1 cells in mice. J Neurosci.

[42] Navarro K.L., Huss M., Smith J.C., Sharp P., Marx J.O., Pacharinsak C. (2021). Mouse anesthesia: the art and science. ILAR J.

[43] Munn B.R., Müller E.J., Wainstein G., Shine J.M. (2021). The ascending arousal system shapes neural dynamics to mediate awareness of cognitive states. Nat Commun.

[44] Zhang L.N., Yang C., Ouyang P.R. (2016). Orexin-A facilitates emergence of the rat from isoflurane anesthesia via mediation of the basal forebrain. Neuropeptides.

[45] Dong H long, Fukuda S., Murata E., Zhu Z., Higuchi T. (2006). Orexins increase cortical acetylcholine release and electroencephalographic activation through orexin-1 receptor in the rat basal forebrain during isoflurane anesthesia. Anesthesiology.

[46] Wang D., Guo Y., Li H. (2021). Selective optogenetic activation of orexinergic terminals in the basal forebrain and locus coeruleus promotes emergence from isoflurane anaesthesia in rats. Br J Anaesth.

[47] Arrigoni E., Mochizuki T., Scammell T.E. (2010). Activation of the basal forebrain by the orexin/hypocretin neurones. Acta Physiol.

[48] Vazquez-DeRose J., Schwartz M.D., Nguyen A.T. (2016). Hypocretin/orexin antagonism enhances sleep-related adenosine and GABA neurotransmission in rat basal forebrain. Brain Struct Funct.

[49] Jones B.E. (2020). Arousal and sleep circuits. Neuropsychopharmacology.

[50] Kelz M.B., Sun Y., Chen J. (2008). An essential role for orexins in emergence from general anesthesia. Proc Natl Acad Sci U S A.

[51] Dong H., Niu J., Su B. (2009). Activation of orexin signal in basal forebrain facilitates the emergence from sevoflurane anesthesia in rat. Neuropeptides.

[52] Trivedi P., Yu H., MacNeil D.J., Van der Ploeg L.H., Guan X.M. (1998). Distribution of orexin receptor mRNA in the rat brain. FEBS Lett.

[53] Soya S., Takahashi T.M., McHugh T.J. (2017). Orexin modulates behavioral fear expression through the locus coeruleus. Nat Commun.

[54] González J.A., Jensen L.T., Fugger L., Burdakov D. (2012). Convergent inputs from electrically and topographically distinct orexin cells to locus coeruleus and ventral tegmental area. Eur J Neurosci.

[55] Manns I.D., Alonso A., Jones B.E. (2000). Discharge profiles of juxtacellularly labeled and immunohistochemically identified GABAergic basal forebrain neurons recorded in association with the electroencephalogram in anesthetized rats. J Neurosci.

[56] Gritti I., Mainville L., Jones B.E. (1993). Codistribution of GABA- with acetylcholine-synthesizing neurons in the basal forebrain of the rat. J Comp Neurol.

[57] Yang C., Thankachan S., McCarley R.W., Brown R.E. (2017). The menagerie of the basal forebrain: How many (neural) species are there, what do they look like, how do they behave and who talks to whom?. Curr Opin Neurobiol.

[58] Cardin J.A., Carlén M., Meletis K. (2009). Driving fast-spiking cells induces gamma rhythm and controls sensory responses. Nature.

[59] Razavi B.M., Hosseinzadeh H. (2017). A review of the role of orexin system in pain modulation. Biomed Pharmacother.

[60] Lee H.J., Chang L.Y., Ho Y.C. (2016). Stress induces analgesia via orexin 1 receptor-initiated endocannabinoid/CB1 signaling in the mouse periaqueductal gray. Neuropharmacology.

[61] ho yc, lee hj, tung lw (2011). activation of orexin 1 receptors in the periaqueductal gray of male rats leads to antinociception via retrograde endocannabinoid (2-arachidonoylglycerol)-induced disinhibition. J Neurosci.

[62] Emam A.H., Hajesfandiari N., Shahidi S., Komaki A., Ganji M., Sarihi A. (2016). Modulation of nociception by medial pre-optic area orexin a receptors and its relation with morphine in male rats. Brain Res Bull.

[63] Yazdi-Ravandi S., Razavi Y., Haghparast A., Goudarzvand M., Haghparast A. (2014). Orexin A induced antinociception in the ventral tegmental area involves D1 and D2 receptors in the nucleus accumbens. Pharmacol Biochem Behav.

